# Testing fractional doses of COVID-19 vaccines

**DOI:** 10.1073/pnas.2116932119

**Published:** 2022-02-07

**Authors:** Witold Więcek, Amrita Ahuja, Esha Chaudhuri, Michael Kremer, Alexandre Simoes Gomes, Christopher M. Snyder, Alex Tabarrok, Brandon Joel Tan

**Affiliations:** ^a^Development Innovation Lab, University of Chicago, Chicago, IL 60637;; ^b^Douglas B. Marshall Jr. Family Foundation, Houston, TX 77002;; ^c^Department of Economics, University of Chicago, Chicago, IL 60637;; ^d^National Bureau of Economic Research, Cambridge, MA 02138;; ^e^Department of Economics, Dartmouth College, Hanover, NH 03755;; ^f^Department of Economics, George Mason University, Fairfax, VA 22030;; ^g^Department of Economics, Harvard University, Cambridge, MA 02138

**Keywords:** COVID-19, vaccines, pandemic, fractional dosing, dose stretching

## Abstract

Due to the enormous economic, health, and social costs of the COVID-19 pandemic, there are high expected social returns to investing in parallel in multiple approaches to accelerating vaccination. We argue there are high expected social returns to investigating the scope for lowering the dosage of some COVID-19 vaccines. While existing evidence is not dispositive, available clinical data on the immunogenicity of lower doses combined with evidence of a high correlation between neutralizing antibody response and vaccine efficacy suggests that half or even quarter doses of some vaccines could generate high levels of protection, particularly against severe disease and death, while potentially expanding supply by 450 million to 1.55 billion doses per month, based on supply projections for 2021. An epidemiological model suggests that, even if fractional doses are less effective than standard doses, vaccinating more people faster could substantially reduce total infections and deaths. The costs of further testing alternative doses are much lower than the expected public health and economic benefits. However, commercial incentives to generate evidence on fractional dosing are weak, suggesting that testing may not occur without public investment. Governments could support either experimental or observational evaluations of fractional dosing, for either primary or booster shots. Discussions with researchers and government officials in multiple countries where vaccines are scarce suggests strong interest in these approaches.

## Significance

Switching to fractional doses could dramatically accelerate vaccination, and clinical evidence suggests that fractional doses of COVID-19 vaccines could be highly effective. However, there is uncertainty about the effectiveness of fractional doses. In this paper, we present the existing evidence and use epidemiological models to quantify benefits under various scenarios. We argue for more experimental or observational data to be collected urgently. Because switching to fractional dosing could dramatically accelerate vaccination, the potential benefits of further testing of fractional doses far outweigh the costs.

Early in the COVID-19 pandemic, the International Monetary Fund estimated that it would cost the world $12 trillion in short-run GDP losses alone over a 2-y period (1). Subsequent estimates including health costs and long-run impacts are much larger (2). Based on these estimates, accelerating mass vaccination by even a month would be worth at least $500 billion (3).

The high value of accelerating vaccination suggests that decisions early in the pandemic to invest in multiple vaccine candidates and in installing manufacturing capacity for vaccines in parallel with research and development, rather than in sequence, had high expected social value, despite risks that individual investments might fail or prove redundant. For example, a rough estimate suggests that the US Operation Warp Speed (OWS) would have paid for itself if it advanced vaccination in the United States by less than a day.* Because the social value of accelerating vaccine availability so greatly exceeds the commercial value to vaccine manufacturers, vaccine capacity investments on the scale of OWS would likely not have occurred without public financing.

While OWS generated sufficient vaccines for the United States, many countries still face supply constraints (5). Similar to at the beginning of the pandemic, pursuing multiple options in parallel that have even a modest chance of accelerating mass vaccination would both have high expected return on investment and promote equity. This could include improving vaccine delivery systems, investing in expanding supply, and exploring options for using existing supply more efficiently, as insurance against a range of plausible scenarios, including against downside risk from new variants that require booster shots, or shocks to the supply chain.

Using lower doses of vaccines is one such option (see, e.g., ref. 6). Fractional dosing has been employed successfully for multiple diseases, including in 2016–2018 when several countries used 1/5 doses of yellow fever vaccine to combat epidemics based on advice from the World Health Organization (WHO) (7). For COVID-19 vaccines, immunogenicity data coupled with a model-based analysis suggest that half or even quarter doses of some vaccines could be almost as efficacious as currently used doses of the same vaccines and more efficacious than other vaccines currently in use. They may also have lesser side effects. Lower doses are already being tested and used for children and as booster shots. Even if fractional doses are less effective than standard doses, our epidemiological analysis suggests that increasing the speed of vaccination would reduce total infections and deaths under a wide range of conditions. Given the large potential benefits, investing in generating evidence on the efficacy of fractional doses and validating processes for delivery at scale has a high expected return. Alternative dosing regimens may be beneficial for both primary and booster vaccination series.

The emergence of variants of concern (VOCs) has shown that outbreaks can occur even in settings where the majority of the population has been vaccinated with highly effective vaccines (8, 9). However, even the less effective vaccines are still very effective at preventing hospitalizations and deaths, even for VOCs. Moreover, when vaccination rates are low (as is the case currently in many low- and middle-income countries), the direct benefits of vaccination far outweigh the indirect epidemiological impacts. This suggests that, where vaccine supply is constrained or VOCs dominate, outbreaks would occur regardless of dose size, but fractional dosing could protect more of the population from severe disease and death.

Strategies to stretch limited vaccine supplies would have been most valuable early in the pandemic. However, there are several reasons why research on optimal dosing is still important. First, many countries still face supply constraints, which may be exacerbated by growing demand for boosters, or supply shocks. Research on optimal dosing is a public good that offers an insurance policy against low supply. Second, lower doses of some vaccines may be more effective than standard doses of other vaccines. Fractional dosing could therefore increase the supply of the most effective vaccines. Third, lower doses of the same vaccine may be superior if they offer comparable efficacy with lesser side effects.

In this paper, we first argue that the tight relationship between neutralizing antibody response and vaccine efficacy, combined with existing evidence on immune response for lower doses, suggests there is a realistic possibility that high levels of protection could be generated by much lower doses, potentially dramatically accelerating vaccination. We then use an epidemiological model to assess the trade-off between efficacy for those receiving vaccines and overall public health impact, and discuss the potential risks of switching to fractional doses. We outline possible designs for gathering more evidence and argue that there is a gap between the social value and commercial incentives for such research, suggesting that it may not occur without public financing. Much of our analysis is not just relevant for COVID-19 but also has implications for future pandemics. Finally, we discuss the implications of the Omicron variant for fractional dosing.

## Potential Efficacy of Lower Doses

**Fig. 1.**
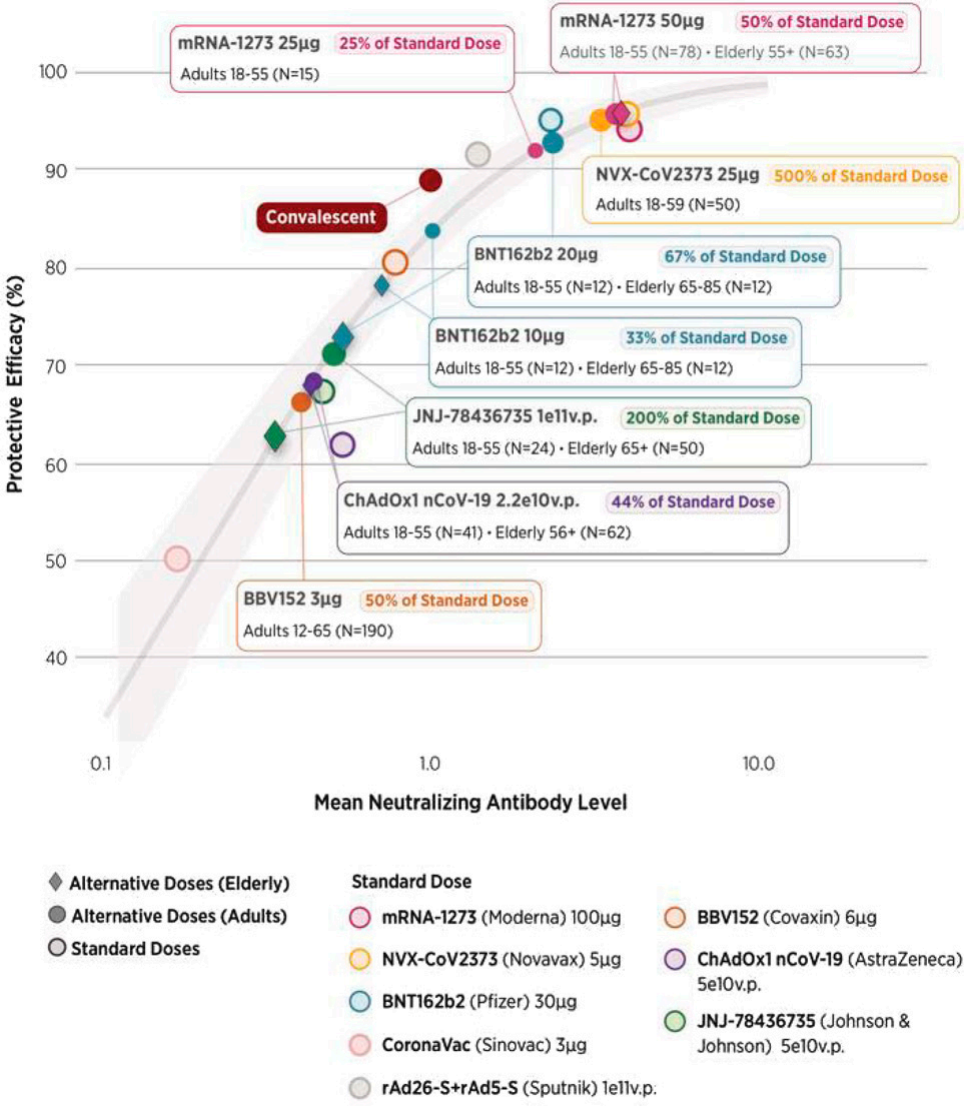
Efficacy associated with mean neutralization levels for fractional doses. The curve follows the model derived by Khoury et al. (10) linking NAb levels (horizontal axis) to protection from symptomatic infection (vertical axis) for standard doses of eight vaccines and in convalescents, with the shaded area corresponding to the 95% CI of the model. Lighter data points represent the mean (normalized) immune response and clinical efficacy against symptomatic infection of specific vaccines (referred to by colors) at standard doses, collected by Khoury et al. (10); response in convalescents is also plotted. NAb levels for vaccines are normalized to those of convalescents, using clinical trial data for each vaccine. We calculate the ratios of mean NAb responses for fractional versus standard doses, using data from clinical trials that tested different doses. We then plot the fractional doses on the immunogenicity–efficacy curve as darker shapes. Doses for the elderly are represented by diamonds, while doses for nonelderly adults (or all adults, where data are not available by age) are represented by circles. For consistency, if multiple age groups are compared, we use the immune response to the standard dose in younger adults to normalize mean NAb levels. We note small sample sizes, typical of early stage trials, and do not include measures of uncertainty.

Efficacy of fractional doses of COVID-19 vaccines has not been tested (except for ChAdOx1 novel coronavirus [nCoV]-19, produced by AstraZeneca, where a low dose–full dose regimen appears to have worked well).^†^ However, phase 1 and 2 clinical trials of various vaccines measured immune response in the form of neutralizing antibody (NAb) titers for different doses (e.g., for messenger RNA [mRNA]-1273, produced by Moderna, four doses were tested). We summarize evidence from trials in *SI Appendix*, section 1 and Table S1. More recently, NAb titers for standard doses were found to be remarkably predictive of efficacy against symptomatic infection in phase 2 and 3 clinical trials (10).^‡^ We used that modeled relationship together with data on NAb titers in fractional doses from dose-ranging studies (*SI Appendix*, Table S2) to derive their predicted efficacy against symptomatic infection (Fig. 1).

Despite the exploratory nature of this approach and the small sample sizes involved, the results strongly suggest that fractional doses of some vaccines produce immune responses that are similar to those produced by larger doses and greater than those produced by standard doses of many other, currently approved vaccines. For example, at 14 d after the second dose, 50- and 25-μg doses of the mRNA-1273 vaccine produced NAb titers not significantly different from the currently used 100-μg doses: The model-predicted efficacy of the first two and the measured efficacy of the standard dose are all within the 90 to 95% range for symptomatic disease. For BNT162b2, produced by Pfizer, 10- and 20-μg doses have model-predicted efficacy of 70 to 85%, versus roughly 95% for 30 μg, the standard dose. Later in the paper, we will discuss evidence generated by more recent trials, as well as ongoing studies of fractional doses.

Many of the phase 2 and 3 trials did not measure efficacy against VOCs, against which the levels of protection from symptomatic disease may be lower. For example, for BNT162b2 and ChAdOx1, real-world data suggest that effectiveness of the standard dose decreased to 88% and 67%, respectively, against the Delta variant (12). We summarize effectiveness data in *SI Appendix*, Table S4. These decreases are also predicted by drops in NAb titers against variants. We summarize NAb data and compare them to effectiveness in *SI Appendix*, section 1.2.

However, all vaccines with available data are highly effective against severe outcomes, even in the presence of VOCs. For example, in the United Kingdom, BNT162b2 and ChAdOx1 were found to be 96% and 92% effective against hospitalization, respectively (13); data from Chile suggest that even CoronaVac (the least effective of the vaccines in Fig. 1) is 86 to 88% effective against hospitalization and death (14). Cell-mediated immunity, rather than neutralizing antibody titers, may be the basis of high levels of durable protection from severe disease (15, 16). Although no clinical data exist, it is therefore reasonable to expect that the decrease in protection against severe disease and death from fractional doses is much smaller than for symptomatic infection.

At this stage of the pandemic, large portions of the unvaccinated population have already been exposed to COVID-19 and acquired some immunity through infection (e.g., refs. 17 and 18). Due to age prioritization and demographic patterns in low- and middle-income countries, the current global population of unvaccinated individuals is also younger, on average, than the world population, and perhaps younger than the populations in which vaccines were originally tested. Recent evidence suggests that previously infected individuals may only need one vaccine dose to be highly protected against reinfection (19). Similarly, because immune responses in younger people are stronger (*SI Appendix*, Table S2), the optimal dose for children and young adults may be lower than for older adults. For example, the United States has approved the use of 10 μg (1/3 doses) of Pfizer for 5- to 11-y-olds, and 3 μg (1/10 doses) are currently being tested (20, 21). Similarly, Moderna is testing 50 μg (1/2 doses) of its vaccine for children (22).

Some clinical data also suggest that fractional doses produce fewer side effects (23). If efficacy is comparable to that of standard doses, and side effects are lesser, fractional doses might be superior to current doses in terms of individual benefit–risk profile.

In discussing health benefits and risks, we have so far focused on primary vaccination series, where more data are available. Optimizing dose size for booster shots could also improve public health outcomes. At the individual level, existing experimental data suggest that a 50-μg-dose booster of mRNA-1273 (half of the standard primary series dose) produces a strong immune response, comparable to peak response after the primary vaccination series with a standard dose (24). Using lower doses would also decrease the strain from boosters on the global supply of vaccines.

In November 2021, the US Food and Drug Administration (FDA) amended the emergency use authorization for Moderna vaccine to include a 50-μg booster for all individuals over 18 y old (25).

## Simulating Public Health Benefits of Fractional Dosing

The traditional research and development process for vaccines is designed to optimize outcomes for the individual taking the vaccine, trading off efficacy and side effects. However, when there is a shortage of vaccines, switching to a lower vaccine dose and vaccinating more people can increase overall public health outcomes, even if vaccine efficacy for the individuals taking vaccines is significantly reduced, since the alternative is to leave more people completely unprotected for longer.

We use an epidemiological model to investigate under what conditions fractional dosing would be optimal at the population level. We simulate vaccination across a range of epidemic scenarios in a modified susceptible–exposed–infected–recovered model with a single epidemic peak (to focus on the immediate impact of vaccination), which accounts for the age-varying effect of vaccination on infections and deaths. Methods are described in *SI Appendix*, section 2.

To fix ideas, we start by considering a base case of a vaccine with 95% efficacy against infection, comparable to efficacy against symptomatic disease of the best vaccines measured in phase 3 trials (*SI Appendix*, section 1). We assume a vaccination rate of 0.25% of the population per day, approximately the recent global median (*SI Appendix*, section 2), and that older individuals are vaccinated first. We consider a range of losses of immune response, which we define through ratios of NAb levels. We then use the model from Fig. 1 to calculate predicted efficacy loss.^§^ We consider the case in which vaccination rates are constrained by supply, rather than demand or distribution, and therefore inversely proportional to dose size (as a fraction of the current dose).

To account for the emergence of VOCs, we also consider a vaccine with 70% efficacy against infection, comparable to reported effectiveness of ChAdOx1 against symptomatic infection with the Delta variant (*SI Appendix*, section 1). We hold other assumptions constant.

As discussed, real-world data for COVID-19 suggest that vaccines have higher effectiveness against severe outcomes than against infection, especially for less effective vaccines and in the presence of VOCs. To address this, we run a third analysis varying efficacy against infection while holding efficacy against death fixed at 95%.

The results for cases with 70% and 95% efficacy against infection are given in Table 1. We find that, if half doses are as efficacious as full doses, then switching would reduce deaths by 22 to 47% for a baseline 95% efficacious vaccine and 20 to 35% for a 70% efficacious vaccine, compared to using a standard dose (the range of outcomes represent different epidemic scenarios). Even if a half dose leads to a fivefold reduction in NAb titers relative to a 95% efficacious full dose, the lower dose would reduce total mortality. For a baseline 70% effective vaccine, the threshold is a 2.5-fold reduction. We show reductions in infections (which are similar in magnitude) and additional scenarios in *SI Appendix*, section 2. Thus, our modeling suggests that, even when new variants dominate and lower-dose efficacy is significantly lower than suggested by Fig. 1, using fractional doses of the more efficacious vaccines would save lives.^¶^

**Table 1.** Deaths averted by switching to hypothetical fractional dosing regimens

NAb ratio (efficacy)	Dose			
	1	1/2	1/3	1/4
Switching from 95% effective full dose				
1.0 (95%)	0	**22 to 47**	**32 to 69**	**37 to 80**
0.8 (94%)	−2 to −1	**21 to 45**	**31 to 67**	**37 to 79**
0.4 (87%)	−12 to −4	**18 to 34**	**28 to 59**	**34 to 73**
0.2 (76%)	−29 to −10	**13 to 22**	**23 to 44**	**29 to 60**
Switching from 70% effective full dose				
1.0 (70%)	0	**20 to 35**	**30 to 52**	**35 to 64**
0.8 (65%)	−6 to −3	**18 to 31**	**27 to 45**	**33 to 57**
0.4 (49%)	−27 to −13	−1 to 15	**17 to 32**	**24 to 40**
0.2 (34%)	−52 to −24	−26 to −5	−12 to 12	−3 to 21

Values are 1−(#deaths with fractional dose/#deaths with standard dose). Ranges correspond to different epidemic scenarios (*SI Appendix*, section 2) from *R* = 0.99 to *R* = 2. Positive values (in bold) favor switching to the lower dose. Vaccination rate is proportional to reciprocal of dose. The standard dose (“dose = 1”) column is included for comparison with fractional doses and to illustrate the magnitude of additional burden of mortality due to various levels of loss of efficacy.

The results for the model varying efficacy against infection are presented in *SI Appendix*, section 3. We find that, at the vaccination rates typical in many low- and middle-income countries, even a vaccine with high efficacy against infection does not prevent large outbreaks, simply because not enough people are vaccinated in time. Moreover, as the recent experience of the United Kingdom shows, it is difficult to stop the spread of Delta VOC even with high uptake of the most efficacious vaccines (8, 9). However, in these settings, accelerating vaccination is still beneficial, as it confers direct protection against hospitalization and death to more people. As shown in *SI Appendix*, Table S4, effectiveness against these outcomes is high for all vaccines where data are available.

## Risks of Using Lower Doses

Our modeling does not consider the rate of immunity loss, which has been established for currently used vaccines (e.g., refs. 26 and 27) and will likely be impacted by modifying dose size. Protection from severe outcomes of COVID-19 may be longer lasting (28). However, even assuming duration of immunity is proportional to dose, it will likely be optimal to improve vaccine coverage in the short term by switching to lower doses and then using future supply as boosters. Moreover, shortages are likely to ameliorate over time as more production comes online, production techniques are optimized, and more countries will have made primary series available to those who seek it, decreasing the infection risk.

Another potential risk is that fractional dosing would increase the probability of emergence of more harmful variants of the virus due to selection pressure during a prolonged period of partial immunity. However, these risks may be sufficiently offset by reductions in the total number of infected individuals, and overall risk of emergence of new variants among vaccinated individuals may be low (29). We provide an overview of recent literature in *SI Appendix*, section 1.3.

Vaccination programs that make use of lower doses could also be criticized as inequitable. However, if there is little efficacy loss and a reduction in side effects, lower doses may actually be superior to standard doses from the standpoint of individuals who would be vaccinated in either case. Even if not, vaccinating a greater number of people with a somewhat less efficacious vaccine is still more equitable than the status quo. Moreover, reduced doses of some vaccines, such as the mRNA vaccines, are likely more efficacious than the standard of care in many low- and middle-income countries. As demonstrated by the analysis depicted in Fig. 1, existing evidence suggests that a half dose of mRNA-1273 may be more efficacious than a standard dose of ChAdOx1, and, similarly, that a half dose of ChAdOx1 could be more efficacious than a standard dose of CoronaVac. Hence, fractional dosing may improve the quality of care by increasing the supply of more effective vaccines. Third, increased supply will cut wait times the most for those who have the longest to wait to receive vaccinations. (For example, doubling the speed of a yearlong vaccination program cuts the wait time by a week for someone 2 wk from the front of the queue, but by 6 mo for the person at the end of the queue.)

Another risk would be that switching to fractional doses could contribute to vaccine hesitancy. However, if lower doses of high-efficacy vaccines are safer, reduce side effects relative to existing standard doses, and are more effective than the low-efficacy vaccines currently used in many countries with short supply, fractional dosing might even be helpful in combating vaccine hesitancy. Surveys suggest that, while vaccine acceptance in low- and middle-income countries is higher than in many high-income countries, side effects remain the most common concern among individuals who remain undecided or opposed to vaccination (*SI Appendix*, section 4). For the yellow fever outbreak in Democratic Republic of the Congo and Angola in 2016, the WHO concluded that the rationale for switching to lower doses was well understood by the target population: While there were questions raised, there was no significant resistance or misinformation specific to fractional dosing observed, and overall uptake was high (98% of the target population) (30).

## Testing Fractional Doses

To date, no regulatory agency or immunization advisory group has recommended fractional dosing for COVID-19 vaccines. The WHO Strategic Advisory Group of Experts interim statement on fractional dosing (10 August 2021) encourages more research (31). However, despite the global shortage of vaccines, high expected value of testing, and promising clinical trial data being available since autumn of 2020, very few studies of fractional dosing have been conducted since. One exception is the mRNA-1273 vaccine, where we are aware of three results suggesting safety, strong immune response to primary and booster doses, and durability of protection in lower doses.^#^

As of November 2021, a few studies of fractional doses are ongoing. We are aware of an observational study of efficacy of primary series with half doses of ChAdOx1 nCoV-19 in Brazil (34) and two randomized trials of immunogenicity comparing low doses with full doses for BNT162b2 (36) and mRNA-1273 (32).^‖^ A randomized evaluation of several low-dose boosters has been conducted in the United Kingdom (37). None of these studies are sponsored by vaccine makers. A platform trial design focusing on impact of fractional doses in previously primed individuals has been announced by Coalition for Epidemic Preparedness Innovations in October 2021 (38).

Large sample size trials of efficacy against disease were needed for the initial regulatory approvals for COVID-19 vaccines. However, given recent advances in establishing correlates of protection against infection (10, 39), data on immunogenicity of fractional doses may provide sufficient evidence for some policy makers (in particular, national immunization advisory groups) to recommend their use in national vaccination campaigns, especially for younger adults or other lower-risk groups. Considering small sample sizes of existing dosing trials and lack of data on protection from VOCs, decision makers are likely to request additional data.

Additional immunogenicity trials can be conducted in a matter of months, at low cost, and even when risk of COVID-19 infection is low (as is the case for the three ongoing immunogenicity trials we cited).** Given this timeframe, immunogenicity trials may be optimal for policy makers looking to address vaccine shortages or improve the safety profile of vaccines in the medium term. In the longer term, randomized trials of efficacy may also offer useful data to the policy makers, but they are more time consuming to run and require much larger sample sizes, which, given recent difficulties in procuring vaccines for clinical research (40), may also contribute to delays.

Alternatively, where short-term supply is limited and infection risk is high, some policy makers may decide to roll out the vaccine dose which offers the best expected public health outcome based on the latest available data and use data from the rollout to assess effectiveness and safety (41, 42). The United Kingdom did something similar when they extended the gap between doses of BNT162b2 and ChAdOx1 nCoV-19 to 12 wk in December 2020 based on limited data (43).^††^ The rollout can be limited to certain areas or to lower-risk age groups. Observational data can then be collected in prospective cohort or case-control studies, used to estimate effectiveness and guide further decisions. In parallel, immunogenicity data can also be collected for a smaller subset of the population. If evidence suggests that changing doses is not effective or if supply increases rapidly, the approach can be adjusted or even reversed, just as the United Kingdom reduced the interval between doses for some adults to maximize protection against the Delta variant. For fractional dosing, reversibility would mean increasing the second dose size or providing booster shots.

Ultimately, the decisions will depend on factors that are unique to each country. Between June and August 2021, we reached out to clinical researchers, decision makers in individual countries, and vaccine manufacturers to better understand prospects for testing fractional doses and changing of current recommendations. There was substantial interest from policy makers and researchers in lower- and middle-income countries. Different groups expressed interest in pursuing different approaches, depending on their countries’ current vaccination rates and expected future supply. In some cases, where the majority of a country’s population might have received a low-efficacy vaccine, policy makers wanted to explore fractional dosing for booster shots. In other cases, where vaccine supplies are very low, they were interested in exploring fractional dosing for primary series. There was also variation in preferred approach to testing, with some policy makers considering proceeding with limited rollouts and collecting observational data, while others looked to first sponsor immunogenicity trials, but all agreed that more data should be collected. We are aware of several more immunogenicity trials that are being planned in middle-income countries, but no efficacy studies.

In addition to testing efficacy and safety of alternative doses, logistical questions regarding their administration will also have to be answered through validation studies. Some vaccines could potentially be used off-label in their current formulations. In that case, advisory groups must assess how many times a vial can safely be punctured and whether smaller volumes of vaccine can be administered consistently and with which syringes. These questions can be answered quickly. In the FDA recommendation for the administration of 50-μg (1/2 dose) booster shots of Moderna (44), and in all of the studies so far, lower doses were obtained from standard preparations of vaccine by drawing less volume into syringes.^‡‡^ This suggests that other vaccines may not need changes to fill and finish processes. Any modifications to supply chains and delivery systems can proceed in parallel to testing.

## Gaps between Commercial Incentives and Social Value of Research on Optimal Dosing

In previous research, we estimated the social value of an additional course of vaccine to be $500 to $1,000 (depending on when it was available), which dwarfs the $6 to $40 price that manufacturers receive in current contracts. We have argued that this gap leads to significant underinvestment by private companies in manufacturing capacity, compared to the social optimum (3). It may also lead to private underinvestment in research on other ways to accelerate vaccination. This suggests that such research may not be carried out without public funding (46).

Although some vaccine manufacturers are sponsoring trials of fractional doses for children and boosters (21, 22), private companies may have little reason to conduct research on fractional dosing for primary series and may even face disincentives. Our outreach to selected manufacturers seems to confirm this. We can see three reasons for this. First, if evidence convinced countries to use lower doses off-label by drawing more doses from each vial, and if public relations concerns prevented manufacturers from increasing the vial price, companies would lose revenue. Second, manufacturing vials with specific doses involves substantial sunk costs. Seeking regulatory approval for a new dose is also a costly and slow process. Manufacturers may prefer to focus on products that can be sold at much higher prices in high-income countries, such as booster shots that have been modified to address variants, or combinations of COVID-19 and flu vaccines. Manufacturers may see testing of lower doses of existing products as interfering with this strategy. Finally, firms face a reputational risk if something goes wrong with a lower dose.

Government investment in accelerating the development of first-generation COVID-19 vaccines created benefits in the trillions of dollars (3). Similar investments in testing fractional dosing could also have extremely high payoffs: For a rough sense of the magnitude of the effect, a simple calculation based on manufacturer’s production targets for 2021 suggests that implementing fractional dosing globally for the most promising vaccines could potentially increase vaccine supply by 450 million to 1.55 billion doses per month in the last quarter of 2021 (*SI Appendix*, section 5).

Even at the government level, no single country internalizes the total global benefit of testing, because the information generated by testing is a global public good. For example, following the United Kingdom’s successful experimentation with a longer delay between first and second doses, many other countries adopted the same dosing schedule (47). Likewise, evidence on fractional dosing could inform decision-making in multiple countries, suggesting a role for global institutions to invest in and coordinate studies.

## The Value of Dose Optimization Late in the Pandemic

Even late in the pandemic, with vaccine supply increasing, research on fractional dosing has a high expected value. Funding research on fractional dosing is a case of investing under uncertainty: Much of the benefit of testing fractional doses comes from their option value in case of possible shocks that might delay supply. These could include the emergence of new COVID-19 variants or other pathogens which could require repurposing of manufacturing capacity, manufacturing or supply chain disruptions or quality issues [such as those experienced by Novavax over the past 6 mo (48)], or other unanticipated factors. Information on fractional dosing will have enormous value in these scenarios. Therefore, even a small chance of delay justifies investment in testing, the cost of which is extremely small in comparison to the potential benefits.

Disregarding potential shocks, our conservative estimate suggests that supply constraints will continue to bind in many countries at least until March 2022 (see *SI Appendix*, section 6 for details). We predict the global shortfall of vaccines using manufacturers’ production estimates for 2021–2022 and simple assumptions about demand, accounting for boosters and assuming that only a fraction of excess supply in high-income countries will be donated to lower-income countries, as has so far been the case.^§§^

The eventual distribution of booster doses in middle- and low-income countries (which has already begun in some places, including Brazil and Russia) may further decrease available supply for primary series vaccination elsewhere. Our estimates also do not account for logistical and political issues that could delay delivery of contracted doses or donations to the countries that need them.

Considering supply to date, these projections should be viewed with caution. For example, Nigeria received 30.2 million doses between March 2021 and November 2021 (an 8-mo interval), 28 million of which came through donations from COVAX or other countries. The country would have to receive 160 million doses in 4 mo in order to meet the predictions from the supply model, or 5 times the amount received so far, in half the time.

Even if there are enough doses to vaccinate the world very soon, fractional dosing has other potential benefits that suggest further research is worthwhile. As we already discussed, lower doses of the same vaccines could also provide comparable protection while reducing side effects, especially for younger or previously infected individuals. Moreover, since lower doses of some vaccines are likely more effective than full doses of many other vaccines, fractional dosing could increase the supply of the most effective vaccines, improving vaccine equity and thus public health outcomes.

Finally, COVID-19 policies will influence future pandemic response. Setting a precedent for testing and potentially implementing dose optimization strategies that can accelerate vaccination is likely to influence decision makers in the future.

## Conclusion

There are risks to using fractional doses, and logistical questions that remain to be answered. However, the reversibility and large potential benefits of fractional doses suggest that testing—in the short term most likely achieved either via immunogenicity studies or rigorously evaluated rollouts of fractional dosing regimens—has tremendous informational value. Clinical data and epidemiological modeling suggest that switching to fractional doses of some COVID-19 vaccines could potentially save lives by accelerating vaccination in countries still facing supply constraints. Fractional doses may be more efficacious than the current standard of care in many countries and may also have lesser side effects.

Given the substantial risks of status quo policies, as recent outbreaks in Southeast Asia and elsewhere have illustrated, the expected value of testing fractional doses is high even with only a modest chance that they will be effective. Furthermore, the risk of changing doses is lower when the unvaccinated population is young or when a higher share of the population has already acquired immunity through infection, as is increasingly the case around the world. If lower doses are found to be effective, they have the potential to save lives. If not, the policy can be reversed. The social value of such testing is significantly greater than the private value, suggesting a role for public funding.

## Coda

In late 2021, emergence of the Omicron variant of SARS-CoV-2 led many countries to accelerate and expand booster programs, due to lower effectiveness of currently available vaccines against Omicron. Some countries have even started to administer fourth doses. This has put substantial pressure on the supply of vaccines, especially mRNA vaccines, meaning that, despite the increased supply discussed above, the share of vaccines going to low-income countries has not been growing in recent months (50). Moreover, if vaccines have to be reformulated against the new variant, the necessary repurposing of vaccine manufacturing will disproportionately affect countries where few people have received primary series vaccinations. As we argued, fractional dosing research offers an insurance against potential shocks to supply such as these.

## Supplementary Material

Supplementary File

## Data Availability

A public repository which can be used to replicate all simulations used in the paper can be found here: https://github.com/wwiecek/covstretch. Previously published data were used for this work (51, 52).

## Footnotes

^*^Between March and September of 2020, the COVID-19 pandemic cost the United States an estimated $16 trillion in long-term losses, approximately $75 billion per day (2). OWS spent $18 billion (4).

^†^Throughout the article, we say “efficacy” in relation to either the effect of vaccines measured in clinical trials or if hypothesized based on a model. We say “effectiveness” to highlight the cases when the effect has been observed in real-world studies.

^‡^In addition to population-level analyses such as the one we use in our model, emerging individual-level data (analyzing clinical trial data for mRNA-1273) also suggest strong association between neutralizing antibodies (at second dose) and risk of disease (11).

^§^We use fold reductions rather than simply reducing efficacy by a set amount, as this allows us to appropriately account for the shape of the curve in Fig. 1. For example, a 2.5-fold reduction in mean neutralization level has a very small impact on efficacy for a 95% effective vaccine (drop to 87% efficacy) compared to a 70% effective vaccine (drop to 49%).

^¶^The base case model assumes that only 80% of the eligible population will get the vaccine, which is largely supported by recent survey data (summarized in *SI Appendix*, section 4). Experiences in multiple (including middle-income) countries suggest that vaccination rates greater than 80% are achievable when supply constraints are eased. As more countries introduce vaccine mandates, we also expect vaccination rates to exceed the initial estimates of vaccine take-up. However, to understand the impact of higher hesitancy, we decrease this value from 80 to 60% and repeat the analysis. While relative benefits of full vs. fractional dosing strategies are attenuated in all cases (due to a decrease in overall benefits from vaccination), we find no qualitative differences in results. The analysis is given in *SI Appendix*, section 4.

^#^The studies find that intradermal administration of a 10-μg dose induces antibody responses comparable to that of convalescent individuals (32); a 50-μg booster shot induces antibody levels similar to or higher than those observed after the primary series with the standard dose (23); and the primary series with two 25-μg doses elicits antibody and cellular protection comparable to that of convalescent individuals, with the cellular protection persisting even after 6 mo (33).

^‖^Preliminary results show that 88% of the subjects with no previous exposure to the virus that received half a dose developed neutralizing antibodies (35). Additionally, NAb titers among subjects that received half and full doses were similar (findings still need to be confirmed by ongoing tests).

^**^Both primary and booster series can be studied. The primary endpoint can be measured within weeks of completing the primary series or receiving boosters, and immunological assays can be conducted in another few weeks. Since small sample size (on the order of 100 subjects per study arm) is sufficient to precisely measure immune responses, it is easy to study many different dosages of the same vaccine and/or subpopulations of interest, for example, further stratifying the subjects by age, immunocompromised status or, in the case of booster studies, vaccination history. Metaanalysis (including hierarchical mechanistic modeling) can be used to synthesize data across multiple studies, including combining new data with previous phase 1 and 2 studies.

^††^Real-world evidence, including observational studies of effectiveness, which we cited earlier, and immunogenicity data, has since emerged supporting the UK decision; this led several countries to delay second doses. Relatedly, existing vaccines were also used at standard dose against the Delta variant, before there was evidence of effectiveness of standard doses against this variant. As viral evolution outstrips the pace of vaccine trials, more such decisions will have to be made under uncertainty.

^‡‡^For 10-μg doses of Pfizer for children, the FDA approved a dilution procedure (45).

^§§^Under different assumptions, estimates from data company Airfinity project that global supply will be sufficient to vaccinate 70% of the global population by May 2022, accounting for donations only from G7 countries and including demand for boosters in this group (49).
