# Supplementary material for: Testing fractional doses of COVID-19 vaccines
Source: Proc Natl Acad Sci U S A. 2022 Feb 7;119(8):e2116932119. doi: 10.1073/pnas.2116932119 (PMC8872706; doi:10.1073/pnas.2116932119)
Supplement: Supplementary File [file pnas.2116932119.sapp.pdf]

Supplementary Information for Testing Fractional Doses of COVID-19 Vaccines

Witold Więcek, Amrita Ahuja, Esha Chaudhuri, Michael Kremer, Alexandre Simoes Gomes, Christopher M. Snyder, Alex Tabarrok, and Brandon Joel Tan

1. Summary of Available Evidence

1.1. Clinical Trial Data

Table S1 lists clinical trials for vaccines discussed in the paper.

In early stage clinical trials, lower dosages of COVID-19 vaccines were often found to stimulate a strong NAb response, at least in non-elderly patients. Evidence on the immunogenicity of a range of dose sizes of each vaccine is summarized in Table S2. Note that in some later trials, such as those for JNJ-78436735 (Johnson & Johnson) and NVX-CoV2373 (Novavax), Phase 3 clinical trials proceeded with the smaller of two dose options tested in early trials after those trials found no statistically significant difference in immune response between the doses.

As discussed in the paper, Khoury *et al.* (*10*) find a "remarkably predictive" logistic relationship between neutralizing antibody (NAb) levels and vaccine efficacy against symptomatic infection, with Spearman ρ of 0.905 (*10*). Authors base their analysis on publicly available data from phase 1-2 clinical trials for NAb data and from phase 3 for vaccine efficacy for subsequently approved vaccines. We assume the relationship between immune response and efficacy holds for all doses tested in phase 1-2 trials and plot additional points based on immune response for all doses of currently used vaccines (Figure 1 in the main text). We use immunogenicity data from phase 1-2 trials that tested different doses (Table S2). Where available, we use the same studies referenced by Khoury *et al.* (*10*). The exceptions were: the 25µg dose of mRNA-1273 (Moderna) (*33*); BBV152 (the vaccine developed and manufactured by Bharat Biotech, sold under brand name COVAXin) (*34*); ChAdOx1 nCoV-19 (AstraZeneca-Oxford) (*14*).

We use the data from those trials to calculate the ratio of mean NAb levels for alternative versus standard doses in each study. In some of the trials multiple age groups were tested, so for consistency we always use in the denominator the NAb levels for standard doses among non-elderly adults (under 55, 59 or 65 years old depending on the study). We then multiply the ratio of alternative versus standard doses by the ratio of standard doses versus convalescents (reported

by Khoury *et al.* (*10*)) for each vaccine to make our estimates of efficacy comparable to those calculated by the authors of the original study.

Our results show that for some vaccines, immune responses associated with high efficacy can be obtained even with much smaller doses. For mRNA-1273 (Moderna), for example, doses 1/2 and 1/4 of the standard both have immune response levels associated with 90-95% efficacy, compared to 94.1% initially reported in phase 3 trials (*35*) for the standard dose. For BNT162b2 (Pfizer) there is no significant decrease in NAb level for a 2/3 dose in non-elderly populations (albeit with a very small sample size), while NAb levels are associated with efficacy between roughly 70% and 85% for other dose-age combinations, compared to 95% initially reported in phase 3 trials (*36*) for the standard dose. For other vaccines, we also sometimes observe unexpected trends, where lower doses lead to NAb levels associated with higher efficacy (e.g., NVX-CoV2373 (the vaccine developed by Novavax and not yet approved for distribution) and ChAdOx1 nCoV-19 (AstraZeneca-Oxford). While these results are not necessarily unrealistic, they may be a consequence of limitations of the modeling approach or of uncertainty inherent in early-stage clinical trials (especially the small sample sizes), or both.

1.2. Viral Variants

Recent studies have found a significant decrease in immune response from vaccines for newer variants such as the Delta variant of concern, first detected in India in December 2020. A summary of effectiveness studies is presented in Table S4. Here, we briefly summarise existing evidence on effectiveness of vaccines against variants.

The purpose of this is two-fold: first, to provide a basic validation of the approach of deriving effect based on reductions in NAb levels we described above; second, to motivate choice of parameters in the simulations.

Wall *et al.* (*4*, *37*) use a live-virus SARS-CoV-2 neutralisation assay to determine NAb titres for different variants in 250 participants from the Legacy study. They report a 5.8-fold reduction in NAb levels after two doses of BNT162b2 (Pfizer) when comparing the wild type to the Delta variant and 2.6-fold decrease when comparing Alpha to wild type. This implies a 2.2-fold reduction between Alpha and Delta (5.8/2.6=2.2).

Other studies report the variation in vaccine effectiveness for different variants. Bernal *et*

al. (*11*) report estimates of effectiveness against symptomatic infection using observational data on vaccinated individuals in the UK and conclude that effectiveness against the Alpha variant for BNT162b2 was 94%, dropping to 88% for the Delta type, for ChAdOx1 nCoV-19 (AstraZeneca/Oxford) the drop is from 75% to 67%. Sheikh *et al.* (*38*) analyse data from Scotland and report a decrease in effectiveness against symptomatic infection from 92% with the Alpha variant to 83% with the Delta variant for BNT162b2, and from 81% to 61% for ChAdOx1 nCoV-19.

Combining both types of data provides some measure of external validation of Khoury *et al.*'s model from Figure 1. For example, according to the model, a decrease in efficacy from 92% to 83% (*38*) is associated with a 2.2-fold drop in NAb levels; a decrease from 94% to 88% (*11*) is associated with a 2.0-fold drop. We can see that these values are comparable to the 2.2-fold drop reported in (*4*). Moreover, focusing on the Delta variant alone, Wall *et al.* report a 2.5-fold drop in NAb levels from ChAdOx1 nCoV-19 when compared to BNT162b2 (*37*). Sheikh *et al.* report a 83% effectiveness for BNT162b2 against Delta while ChAdOx1 nCoV-19 is 61% effective. In Khoury *et al.*'s model, this is associated with an 3.1-fold drop in NAb levels, once again comparable to the decrease reported in observational studies.

1.3. Immune Escape Risk

While dates of earliest documented samples for all six WHO variants of concern (Alpha, Beta, Gamma, Delta) or interest (Mu, Lambda) range from May 2020 to January 2021 (Lambda variant in Colombia), that is, before widespread vaccination campaigns, one serious concern about dose sparing strategies for COVID-19 is that they might contribute to development of more dangerous variants of SARS-CoV-2 (*39*). (This has been discussed especially in the context of delaying vaccination with the second dose, which several countries decided to do in early 2021.) In particular, some experts believe that dose sparing can contribute to immune escape risk due to large number of partially immune vaccinated individuals (*40*).

However, as others have pointed out, in the case of SARS-CoV-2 characteristics of virus and data on response to first doses (especially reduced viral loads in infected individuals) of currently available vaccines suggests prioritising "breadth of vaccine coverage, rather than strength of immunity" (*41*). Similar conclusions have been presented by both a perspective article and a recent

modeling study assessing epidemiological impacts of immune escape (*42*, *43*). The New and Emerging Respiratory Virus Threats Advisory Group (NERVTAG), a scientific advisory group to the British government, analyzed earlier this year (*44*) the risk of immune escape and concluded that although the risk of immune escape from delaying a second dose should be considered, it is likely small, especially in comparison to other sources of immune escape such as therapeutics and natural infection. Recent non-peer-reviewed article also suggests that the mutation rate of the Delta variant has also been lower in countries with higher vaccination rates (*45*).

Since likelihood of evolution of more dangerous variants depends both on number of individuals immune (leading to higher evolutionary pressure) and number of infections (leading to higher number of mutations), assessing the absolute risk is difficult and multifactorial (also dependent on e.g. stringency of non-pharmaceutical interventions). Thus we do not model these risks here. Regardless of the assessment of absolute level of risk there seems to be a broad agreement between different authors that more equitable vaccine distribution would help alleviate the risk.

2. Epidemiological Simulations

2.1. Epidemiological Model

Since the evidence on alternative dose efficacy is not dispositive, we model the potential impact on the pandemic of a range of efficacy levels using a standard epidemiological model. The model we use extends the canonical susceptible-exposed-infectious-recovered (SEIR) model, which is widely used in mathematical epidemiology to characterize the spread of an infectious disease in a closed population (*46*, *47*). The SEIR model assumes individuals flow between disease and vaccination states over time, with sizes of population in each state changing according to a set of differential equations. We extend the canonical SEIR model to allow for death and vaccination (which is ineffective for some individuals), yielding the following equations:

$$\dot{S}_i(t) = -\lambda_i(t)S_i(t) - v_i(t)\delta_i\tilde{S}_i(t) \tag{1}$$

$$\dot{E}_i(t) = \lambda_i(t)[S_i(t) + N_i(t)] - \gamma' E_i(t) \tag{2}$$

$$\dot{I}_i(t) = \gamma' E_i(t) - \gamma_i'' I_i(t) \tag{3}$$

$$\dot{D}_i(t) = p_i \gamma'' I_i(t) \tag{4}$$

$$\dot{R}_i(t) = (1-p_i)\gamma'' I_i(t) - v_i(t)\delta_i \tilde{R}_i(t) \tag{5}$$

$$\dot{P}_i(t) = v_i(t)\delta_i \left[e\tilde{S}_i(t) + \tilde{R}_i(t) \right] \tag{6}$$

$$\dot{N}_i(t) = v_i(t)\delta_i(1-e)\tilde{S}_i(t) - \lambda_i(t)N_i(t). \tag{7}$$

Dots denote derivatives with respect to time. Uppercase letters denote population compartments (i.e., the fraction of the population in a given state): S for susceptible, E for exposed (individuals carrying the virus, but who are not yet contagious), I for infectious, R for recovered, D for dead, P for protected by vaccine, and N for vaccinated but not protected. The population is divided into G age cohorts, indexed by $i = 1, \ldots, G$, with respective sizes n_i. Subscripting compartments by i allows for different epidemic evolution across age cohorts. Tildes denote the size of the compartment in proportion to both compartments receiving vaccines (susceptible and recovered) i.e.,

$$\tilde{S}_i(t) = \frac{S_i(t)}{S_i(t) + R_i(t)} \tag{8}$$

$$\tilde{R}_i(t) = \frac{R_i(t)}{S_i(t) + R_i(t)}. \tag{9}$$

Figure S1 depicts the population flows between the compartments described in equations (1)–(7). For simplicity, we consider the case of either a single dose vaccine or a two-dose vaccine where efficacy does not change between doses. Lowercase letters denote model parameters governing the evolution of compartments. All parameters except e are age-specific, as denoted by subscript i. γ' and γ'' are, respectively, the hazard rates of moving from exposed to infected and from infected to recovered or dead. These are estimated as the reciprocals of the durations of the virus's incubation period and of the infectious period, respectively. The rate of new infections equals $\lambda_i(t)$, described in further detail below. Parameter p_i is the mortality risk. Vaccine efficacy, denoted e, is the probability the vaccine protects from infection. The model makes no distinction between the vaccine's *efficacy* (performance measured in clinical trials) and *effectiveness* (performance in practice in the population); e is used to denote both interchangeably. We assume recovered individuals (compartment R) are perfectly protected by vaccination and that exposed or infectious individuals (compartments E and I) are not vaccinated.

To account for vaccine prioritization, we introduce an indicator variable $v_i(t)$, switching from 0 to 1 on the day age cohort i becomes eligible for vaccination and to 0 again at the point where all willing members of the cohort have been vaccinated. Reflecting common practice, we assume older cohorts must finish vaccinations before the next cohort becomes eligible. When $v_i(t) = 1$, age cohort i is vaccinated at a constant rate δ_i, drawing on a continuous stream of vaccine production from a given capacity. To keep track of cumulative doses distributed, we introduce the auxiliary compartment V, where $\dot{V}_i(t) = \delta_i v_i(t)$, and where $V(t)$ is the proportion vaccinated in the entire population.

The rate of new infections, $\lambda_i(t)$, depends on the number of daily contacts a susceptible individual has with currently infectious individuals. To reflect differences in interaction across age cohorts, we use a contact matrix C, where entry $c(i,j) \geq 0$ denotes the number of contacts made by an individual in cohort i with an individual in cohort j. To derive the proportion of each age group infected at time t, each contact is scaled by the probability of virus transmission on contact, q, and probability that the contacted person is infected, $I_j(t)$, yielding

$$\lambda_i(t) = q \sum_{j=1}^{G} c(i,j) I_j(t). \tag{10}$$

For a given C, q can be adjusted to match any desired reproductive number $\mathcal{R}$ for the virus (i.e., the number of secondary cases produced by a single infection).

The initial conditions of the system (1)–(7) require specifying the proportion of the population that is susceptible $S(0)$, immune $R(0)$, and infectious $I(0)$ at the outset of the epidemic. We generally take $I(0)$ to be small and for simplicity take $E(0) = I(0)$. We assume that the proportion of each age cohort in each initial compartment is the same as in the overall population.

2.2. Initial Conditions

We run simulations for three illustrative epidemic scenarios designed to span a range of cases. The *slow decrease* scenario sets the initial effective reproduction rate to $\mathcal{R} = 0.99$ and initial infectious proportion to $I(0) = 1\%$. Since we assume 20% of pre-existing protection in people aged 20 and over, the initial effective reproductive number $\mathcal{R}$ is lower than $\mathcal{R}_0$, the basic reproductive number in a fully susceptible population. The *slow decrease* scenario may capture a situation in

which non-pharmaceutical interventions (NPIs) are introduced following an epidemic wave but are only effective enough to decrease cases slowly. The *slow growth* scenario sets $\mathcal{R} = 1.1$ and $I(0) = 0.5\%$, perhaps reflecting a situation in which NPIs are not effective enough to prevent a subsequent wave of infections, such as the one experienced by the United States in late 2020. The *fast growth* scenario sets $\mathcal{R} = 2$ and $I(0) = 0.1\%$, e.g., a case when a new virus strain suddenly emerges, thwarting previously effective NPIs (such as the one observed in the United Kingdom in December 2020, or the emergence of the P.1 variant in Brazil in late 2020 (*48*). In both growth scenarios, $I(0)$ is adjusted so that the peak of infections occurs three to four months from the start of vaccinations.

We choose parameters for initial immunity that broadly reflect the state of the COVID-19 pandemic in early 2021. We assume 20% of people aged 20 and over have immunity acquired from infection, leaving 80% susceptible. To reflect the lower clinical case rate in the younger population (*49*, *50*), we assume only 50% of under 20s are susceptible.

2.3. Parametrization

Each simulation runs for $T = 365$ days. This is sufficient time for the epidemic to die out in the scenarios considered but, we assume, not long enough for unmodeled factors to come into play, such as the alleviation of supply constraints with expanded capacity or the waning of vaccine protection from initial doses, perhaps warranting booster shots. Similarly, we assume that there is no natural loss of immunity (no flow from recovered to susceptible) during the simulation period. While the simulation period is 1 year, in all epidemic scenarios most infections happen within the first months of the period. For example, for the fast growth scenario the share of infections that happen within the first 4 months is approximately 100%, while for the slow growth scenario this falls to approximately 50%.

We use a social contact matrix $c(i, j)$ based on a large cross-country study of contacts between different age groups, primarily in European countries (*51*). Our matrix is therefore more representative of high-income countries, but we are not aware of comparable data on social mixing in low-income countries. Cohort size (n_i) and mortality risk (p_i) for different age cohorts is consistently based on data for high-income countries. Throughout the age distribution, the risk of death from COVID-19 increases rapidly with age, about three-fold per decade (*52*).

The model assumes that contact frequencies are independent of infection risk, precluding behavioral changes in response to changes in infection risk as the epidemic progresses. We also assume that epidemics always have a single peak and fade out when the virus's effective reproductive number satisfies $\mathcal{R} \leq 1$, which happens when a sufficiently high fraction of population is protected, either by vaccination or recovery from natural infection.

The base case for vaccination is a 95% effective vaccine, when used as tested in Phase 3 trials (standard dosing, with a delay between two doses). We assume that those under 20 (constituting 22% of population in our base case simulations) receive no vaccination. To account for vaccine hesitancy, we assume 20% in each age group refuse vaccination. We assume that the vaccine becomes effective 10 days after it is administered. We achieve this by treating vaccinated compartments in the model as "effectively vaccinated". Hence if vaccinations in a given age group start of day t_1 and end of t_2, we start the flow into vaccinated compartments on date $t_1 + 10$ and stop it on $t_2 + 10$.

As of early May 2021, countries were vaccinating at a median rate of approximately 0.25% of the population per day (*53*), our base case immunization speed. At the high end, countries such as the United Kingdom, United States, Canada, Chile, and Israel have all managed to vaccinate at rates well above 0.8% of the population per day; however, the median global rate of vaccination (as of September 27, 2021) is approximately 0.25% similar to what was observed in May 2021 when we ran our simulations (*53*). Thus, at a global level, supply rather than delivery logistics or demand (e.g., vaccine hesitancy) seem likely to constrain full vaccination well into 2022, and perhaps for considerably longer. This is also supported by manufacturing forecasts (*54*). Therefore, we can expect vaccine supply to be a constraint during the critical infection period of our simulation.

Accordingly, our model is intended to apply to contexts in which vaccination rates are constrained primarily by the available supply. While this may not apply for some countries, this seems broadly to be the case globally in the sense that increases in vaccine supply could effectively be used. The model could be extended to consider other scenarios where, for example, delivery constraints might at some point be binding.

Additionally, while we treat efficacy as a scalar, in reality it is multidimensional: vaccines may differ in efficacy against different variants, in duration of protection, or in their protection against infection and disease.

2.4. Simulation Method

We generate a simulation run for each configuration of parameters by finding the deterministic solution of the differential-equation system consisting of these equations (1)–(7) using standard numerical methods. We solve all differential-equation systems using the odin package, version 1.0.8, and generate exhibits using R, version 4.0.2. All code used in this project is available at https://github.com/wwiecek/covstretch.

Figure S2 illustrates the evolution of vaccinations and infections for the various epidemic scenarios and vaccination rates analyzed. With no vaccination, we find that from 8% (slow decrease scenario) to 55% (fast growth scenario) of the population get infected during the simulation period. Individuals aged 20 to 49 are responsible for between 55% and 59% (depending on the scenario) of all infections, assuming no vaccine. This is consistent with recent estimates (*55*) that three quarters of infections in the US originated from individuals in that age bracket (albeit in a period with school closures).

The outcome variables for our simulations are the burden of infection, defined as the proportion of the total population that develop new infections during the simulation period, and the burden of death, defined as the proportion of the total population that die during the simulation period.

2.5. Simulations

We consider the case in which the pace of immunization is subject only to a supply constraint, therefore the vaccination rate is proportional to the reciprocal of dose size. So, for example, using half rather than full doses would double the vaccination rate. We analyze the impact of alternative dosing on the burden of infections and deaths while varying three variables: dose fractions, efficacy reductions associated with moving to alternative dosing, and epidemic scenarios.

We run scenarios with two different baseline assumptions for vaccine efficacy with full dose vaccine efficacy. First we assume that a full dose has 95% efficacy, compatible with the verified efficacy of mRNA vaccines. Second, to account for the overall drop in effectiveness against some viral variants we repeat our analysis assuming a 70% efficacy for full doses. (This case can also be used to evaluate less effective vaccines in general.)

In order to make the 95% and 70% cases comparable, we define alternative doses efficacy in terms of relative reduction in NAb levels compared to the baseline. To do this, we follow

the previously-cited model by Khoury *et al.*. This allows us to make a "fair" comparison, since comparable fold-reductions in NAb titres will have different impacts on efficacy, due to the concave shape of the curve in Figure 1 in the main text. For example, if starting from the base case of 95% efficacy, a 5-fold reduction in NAb titres is associated with a drop of efficacy to 76%. If starting from 70%, a 5-fold reduction in NAb is associated with efficacy of 34%.

We use ratios of 1, 0.8, 0.4 and 0.2 (1.25, 2.5, 5-fold reductions respectively) to derive the new results. For comparison with measured data, the NAb ratios for fractional doses among non-elderly adults range from 0.43 (1/3 dose of BNT162b2) to 0.91 (1/2 dose of mRNA-1273), the exact values are presented in Table S2. Therefore, despite the exploratory nature of this approach, we find it illustrative to consider a ratio of 0.8 (1.25-fold reduction) with 1/2 dose and a ratio of 0.4 (2.5-fold reduction) with 1/3 dose to guide intuition on expected impact of using alternative doses. 5-fold reduction may correspond to using much lower doses than what was previously tested in dose ranging studies. The results are shown in Figure S3 and summarised in Table 1 of the main text.

We find that for a 95% efficacious vaccine the burden of mortality relative to status quo (risk ratio) is 0.55-0.79 with 1/2 dose and 0.8 NAb ratio and 0.41-0.72 with 1/3 dose and 0.4 NAb ratio, suggesting large benefits of switching to fractional dosing. The ranges correspond to different epidemic scenarios. With 70% baseline efficacy we find a higher but still lower than the status quo relative burden, of 0.69-0.82 with 1/2 dose and 0.8 NAb ratio and 0.68-0.83 with 1/3 dose and 0.4 NAb ratio (drop to 49% efficacy).

3. Differential Vaccine Impact on Mortality and Infection

The initial SEIR model assumes that efficacy of the vaccine against mortality was the same as against infection. However, observational data for multiple vaccines (including mRNA) suggests a differential impact on deaths and infection (and therefore transmission). Therefore we modify the model by adding extra compartments, allowing for differential efficacies against infection and death. A reproducible version of this simple calculation are available in the code repository we referenced earlier.

Let us focus on reducing mortality as the primary objective of a vaccination program and use the fast growth scenario from earlier simulations. In an extreme (and purely theoretical) case,

vaccines have no impact on infection, while providing very good protection against death. In other words, there are no indirect benefits of vaccination and herd immunity can never be achieved. Conversely, when the impact on infection is high, indirect benefits eventually start to outweigh the direct ones.

However, in the current pandemic setting the indirect benefits also depend on speed of vaccinations in relation to infection risk. If only a low proportion of the population can be vaccinated during the exponential growth phase of the epidemic, the impact of infection is low. We illustrate this in Figure S4, where we assume 95% efficacy against mortality and varying efficacy against infection from 0% to 95% (differently coloured lines) and speed of vaccination (x axis). For simplicity we use the fast growing epidemic scenario, but the overall result carries across all scenarios.

We find that at lower vaccination speeds like 0.1 to 0.25% per day (similar to the speed in many lower and middle income countries) the direct effects will outweigh indirect effects. This can be seen in the right panel of Figure S4 (mortality rate), where the lines (corresponding to different levels of sterilising immunity) do not diverge until higher vaccination rates (>= 0.50%) are reached. For example, at 0.25% vaccinated per day we have 54% infected if there is no impact on infection and 45% if the level of protection is 95%. In terms of mortality, we find 17 deaths per 10,000 if there is 50% efficacy against infection, 16 if 95%, and 20 if 0%. We should note that the absolute benefits are very sensitive to the assumption of how far in the future the peak of infections is: here we assume that it is about 3 months as per the fast epidemic growth scenario depicted in Figure S2.

4. Increase in Vaccine Supply from Fractional Doses

While it is hard to predict with precision the increase in supply resulting from the adoption of alternative doses, we present a range of estimates based on the projected supply in 2021 for some of the main vaccines being currently distributed. The results are shown in Table S3.

We use the best projections currently available for vaccine supply in 2021. This includes data from press releases (*56*, *57*) and third-party publications (*58*, *59*) when up-to-date information directly from the manufacturers is not available. This leads to an expected supply for 2021 of 3 billion doses for BNT162b2 (Pfizer), 800 million doses for mRNA-1273 (Moderna), and 2.1 billion doses for ChAdOx1 nCoV-19 (Oxford/AstraZeneca).

We combine the projected supply with the number of doses already delivered according to official statements from vaccine developers UNICEF (*60–62*) (as of September 3, 2021). We subtract doses delivered from projected supply and assume that the remaining quantity will be delivered uniformly during the remaining months of the year. Based on these values, we estimate the number of extra doses that would be generated with the adoption of alternative dosing regimens, as shown in Table S3.

The dosing regimens represented here capture a range of scenarios with varying degrees of optimism. We include only dose sizes that demonstrate NAb levels correlated with high efficacy or comparable to the efficacy of the standard dose in our initial analysis (Figure 1). We observe that for the scenarios considered here, it is possible to produce 450 million to 1.55 billion extra doses per month in the last quarter of 2021.

5. Global Vaccine Supply

5.1. Existing Estimates

A recent analysis by UNICEF estimates global manufacturing capacity of 41 billion doses in 2022, of which 22 billion is for currently approved vaccines (*63*). A shorter-term analysis by Airfinity (dated September 5th, 2021), using production-facility data to focus on production rather than capacity, projects that the global vaccine stock will reach levels sufficient to vaccinate 80% of the global population above the age of 12 by the end of 2021 (*54*).

UNICEF's projections of manufacturing capacity provide an optimistic view on vaccine supply. Their estimates are based on planned capacity as reported by manufacturers, which may never be fully realized. UNICEF acknowledges that "the capacity does not account for vaccine probability of success and may project a highly optimistic view of the potential supply" (*63*). Airfinity's projections use production-facility data instead of reported capacity to address this issue. However, these estimates may still be overly optimistic. First, Airfinity's projections include Novavax, which has not yet been approved by major regulatory agencies. Second, they do not consider potential production shocks as all approved vaccines are assumed to be produced at a steady rate, based on current levels, and face no delays in production. Third, the use of doses as boosters and wastage of vaccines are also not considered. These assumptions have implications on the global balance between supply and demand.

Whatever the global supply, several other arguments can be offered for the per-capita supply within certain countries lagging the global average. Airfinity's projections treat vaccines as exchangeable and do not account for efficacy differences or logistical issues such as differential cold chain requirements, which may make the use of some vaccines less feasible in some countries. A high percentage of doses are contractually committed to a small subset of countries (*64*). Therefore focusing on aggregate global supply estimates alone can create an overly optimistic projection of when all countries (in particular low-income countries) will have adequate vaccine supply.

Some projections, e.g. by the World Bank and IMF-WHO, adopt a country-based approach (*64*, *65*). The World Bank's estimates project administration of doses and are based on current delivery schedules for COVAX, bilateral deals, and dose sharing agreements. For LICs and LMICs these estimates are significantly more pessimistic than Airfinity's analysis would suggest. For example, Kenya, Nigeria, and Ethiopia are expected to vaccinate only 21%, 20%, and 16% of their population by the end of 2021, respectively, according to the World Bank analysis. However, World Bank's projections do not account for potential future donations or potential new bilateral deals between LMICs and vaccine manufacturers. Additionally, they project dose administration, not deliveries or production. The IMF-WHO Covid-19 tracker follows a similar methodology, but includes all current commitments of bilateral deals, COVAX and donations without taking into account a timeline for delivery. Their estimates suggest that vaccines secured to date (even including those arriving after December 2021) will be sufficient to fully vaccinate only 28.5%, 25%, and 16.5% of the population of Kenya, Nigeria, and Ethiopia.[1]

5.2. Supplementary Analysis of Global Supply

In order to account for both possible future donations and likely demand for boosters, we use a simple country-level model to predict how long it will take all countries to obtain enough supply to vaccinate 80% of their population aged 14 or older, allowing for differential access as a function of income level.[2] We predict vaccination rates from October 2021 onwards, using data on current

[1] This assumes all individuals receive vaccine regimes that require two doses for full vaccination.

[2] Airfinity's projections assume all unused doses are donated but do not account for boosters. The World Bank's estimates include demand for boosters but consider only limited donations based on current dose sharing agreements. We consider a realistic demand for boosters as well as all the excess supply from HICs. As detailed further in the text, we apply a reduction factor to the remaining supply from HICs since we assume countries will retain some for their own future use, and some doses will be wasted.

vaccination levels to project future demand for both booster and primary doses in each country.

For primary vaccination, we assume demand consists of the difference between current doses administered and the number of doses required for countries to achieve the target of 80% full vaccination of population aged 14 or older. For boosters, we assume all vaccinated individuals in HICs, as well as those not in HICs who received a primary series of Sinovac, CanSino or Sinopharm, will eventually receive boosters, resulting in a total demand for boosters of approximately 2.3 billion doses. This corresponds to roughly 30% of the global population receiving booster shots. We assume a constant monthly rate of supply over the period of our analysis (6 months), or approximately 380 million booster deliveries per month.

Forecasted monthly supply combines COVAX estimates and non-COVAX doses based on Airfinity's projections. For COVAX, we assume doses are distributed proportionally to each country's population size (excluding high income countries), until 20% of the population is fully vaccinated, reflecting COVAX's allocation mechanism. This accounts for 1.1 billion doses from October 2021 to the end of March 2022. For non-COVAX supply (production estimates from Airfinity minus COVAX), we distribute available doses proportionally to country-level demand for primary vaccination series, up to the point where all countries have administered the primary series for 80% of their population aged 14 or older. This results in a total of 2.4 billion doses for primary series, which would become available over the next 6 months. We assume strict prioritization according to countries' income levels. Our model implicitly accounts for donations by allocating all of non-COVAX supply and allowing countries to receive leftover doses from higher income groups. We assume that only 50% of doses not used by HICs will be used in lower income countries during the modeled period (consistent with projections from Airfinity for excess supply in G7 countries (*66*)).

We find that all countries will reach adequate supply to vaccinate 80% of the population older than 14 years old[3] by the end of February 2022. We note that these estimates only consider a supply perspective, not the administration of doses once supply is available.[4] It also depends on richer

[3]We use data from Our World in Data (OWiD) to derive country-level demand, which groups together individuals aged 15-24. OWiD is our preferred source as it is compatible with other datasets we use (e.g. on current vaccination trends).

[4]In contrast to the estimates presented in Section 4, the data sources used here do not break down projections by vaccine manufacturer. For this reason, we are not able to estimate how adopting fractional dosing approach would impact the forecasts presented in this section.

countries moving forward with vaccine donations, which they may or may not do. With reduced or delayed donations of vaccines that HICs have procured, repurposing of production facilities for new vaccines against variants of concern, increased demand for boosters (e.g. in South Asia) and also with vaccination of younger children, which seems imminent, this date could be pushed further back.

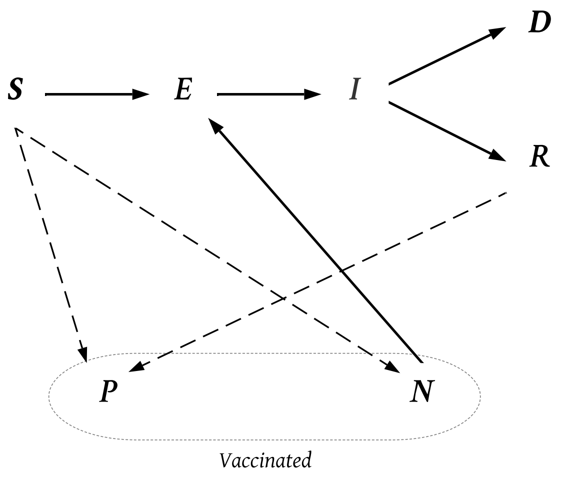

Figure S1: Compartment Flows in Epidemiological Model. Model described by Equations (1)–(7). Solid black lines reflect virus model and dashed lines vaccination. The full model has separate compartments for each age group i.

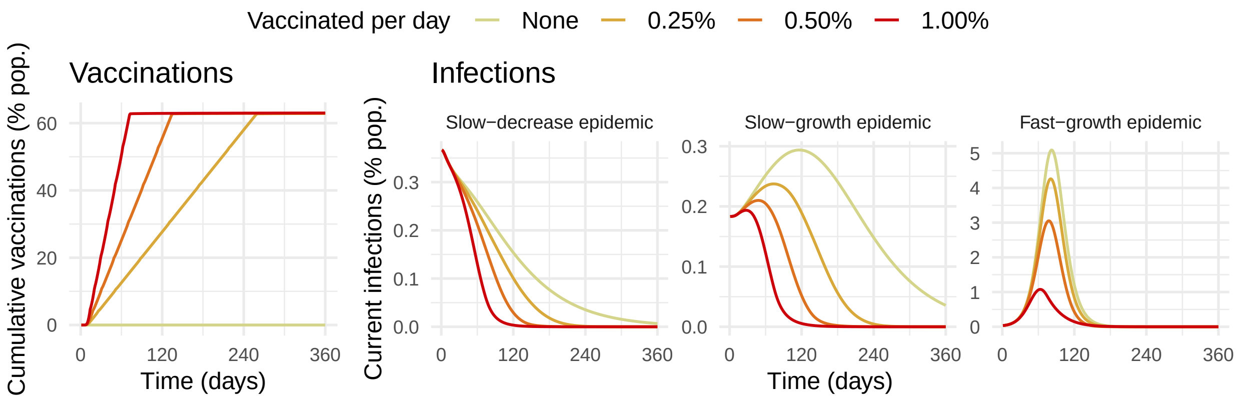

Figure S2: Evolution of Vaccinations and Infections under Baseline Epidemic Scenarios. Colors indicate different vaccination rates δ with a 95% efficacious vaccine. While the population is vaccinated at a constant rate, age prioritization leads different age cohorts to start being vaccinated at different times. Cohorts aged 20 and above achieve 80% vaccination coverage by time $T = 365$ days. Please note that the scales of the vertical axes vary according to the epidemic scenario.

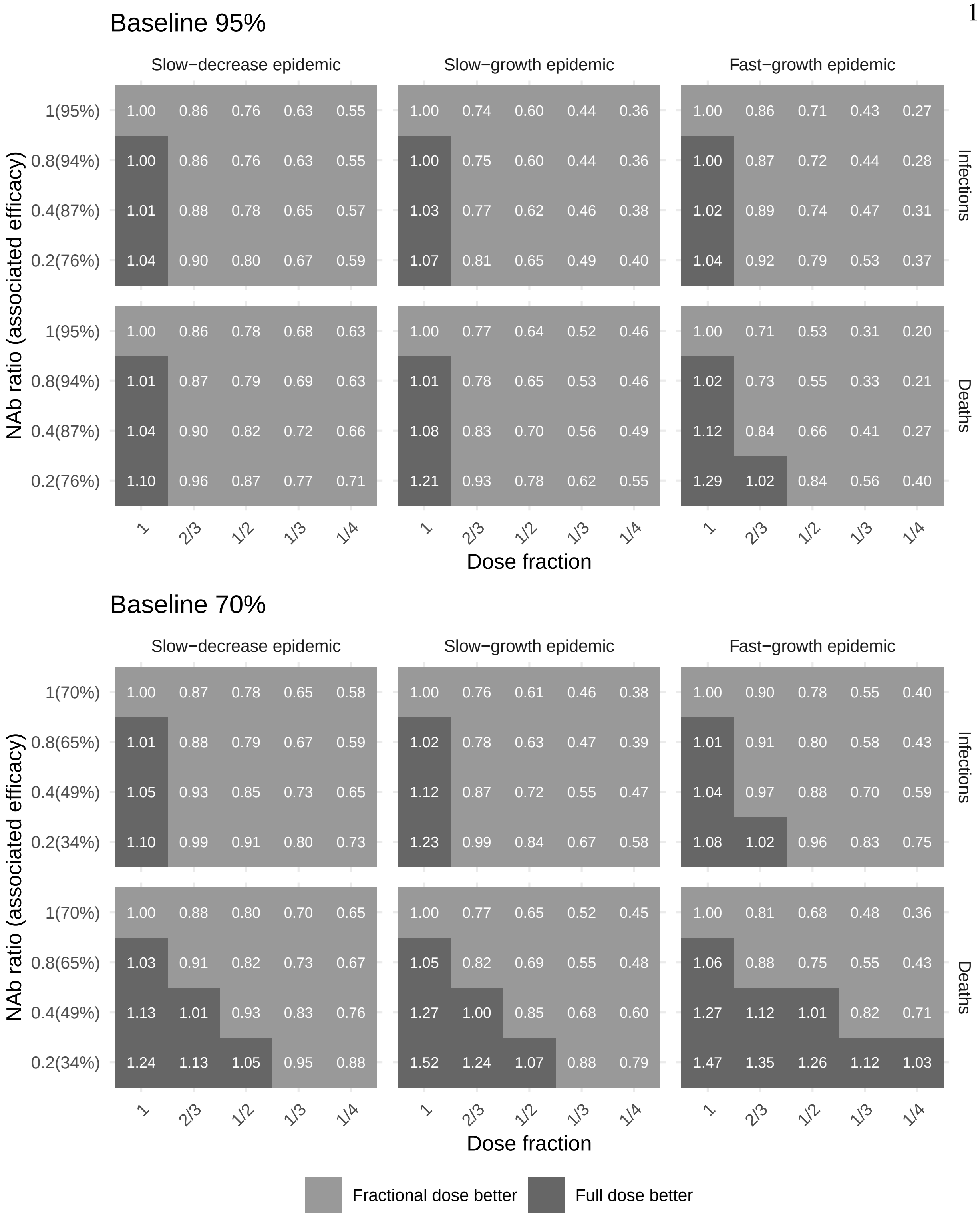

Figure S3: Burden Averted under Full Relative to Alternative Dosing for Different Baselines Entries are burden ratios; greater than 1 (emphasized by darker background) favors full dosing and less than 1 (emphasized by lighter background) favors alternative dosing. Each tile represents a different combination of epidemic growth, level of efficacy of alternative dose, and size of alternative dose, proportional to reciprocal of vaccination rate. The upper figure represents the case for 95% efficacy baseline and the bottom figure represents 70% baseline efficacy. The labels on the vertical axis represent the NAb ratio in relation to baseline followed by the vaccine efficacy estimated using the model by Khoury *et al.* (*10*).

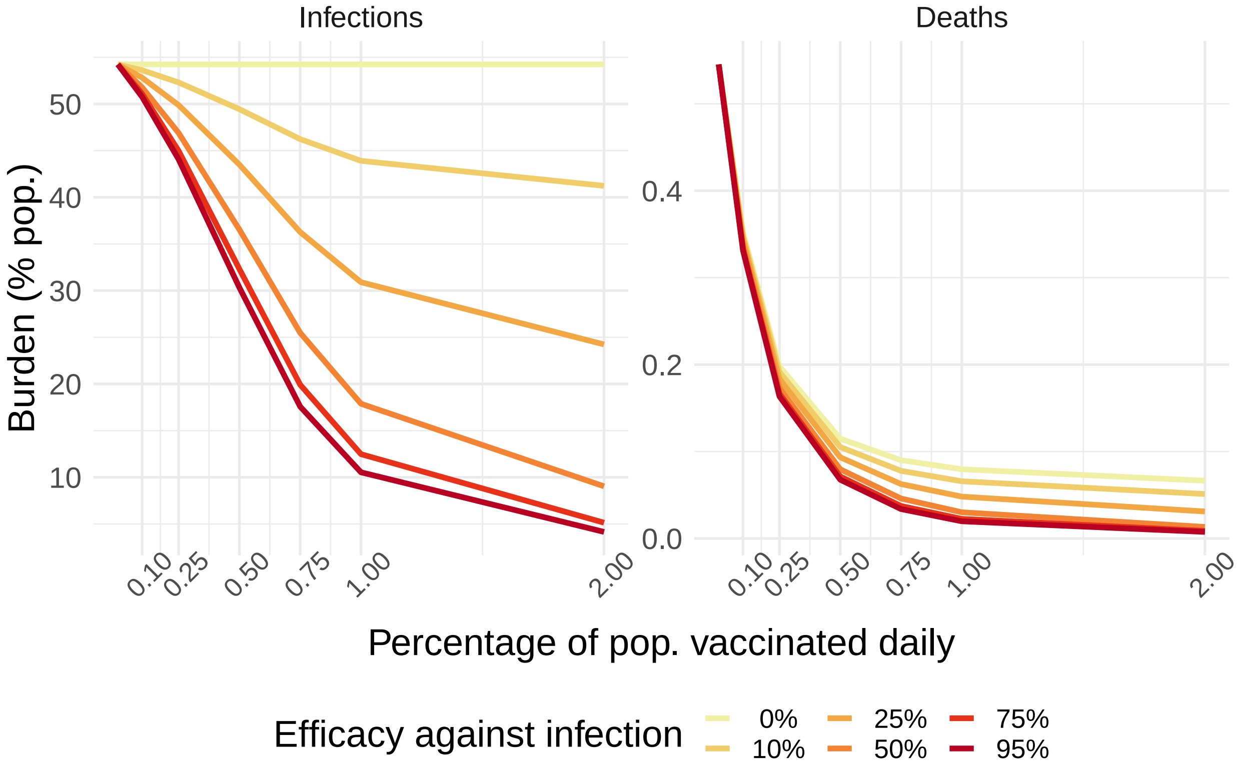

Figure S4: Burden of Infections and Deaths Depending on Efficacy Against Infection. We assume 95% efficacy against mortality; different levels of protection from infection are given by differently coloured lines. Horizontal axis varies vaccination speed.

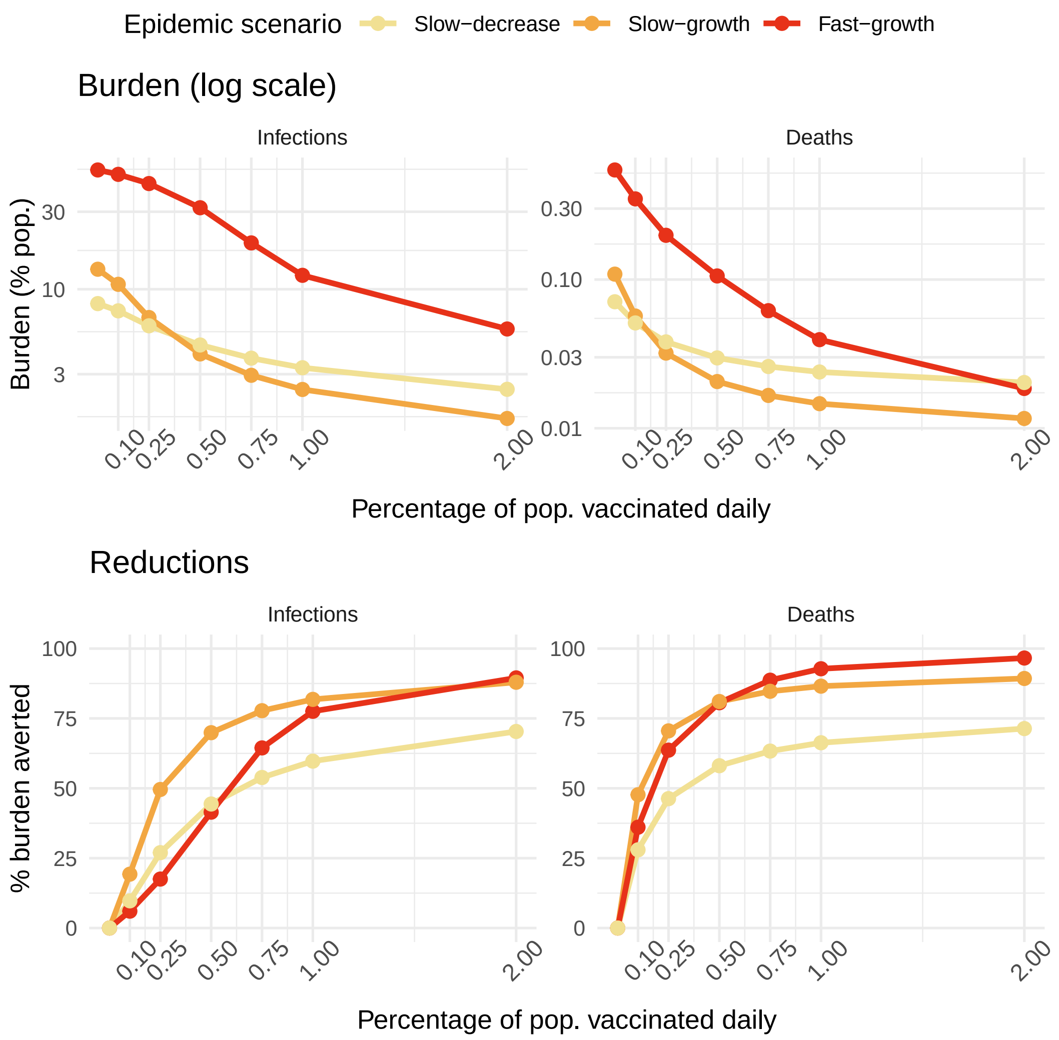

Figure S5: Benefits of Faster Vaccination. The top panels show simulation results for burdens under various vaccination rates for the fast growth epidemic scenario. The bottom panels show simulation results for the percentage reduction in burdens relative to no vaccination. In the left panels, we refer to the burden from infection, while in the right panels we refer to the burden from death. In all scenarios we assume a 95% efficacious vaccine and sequential age prioritization.

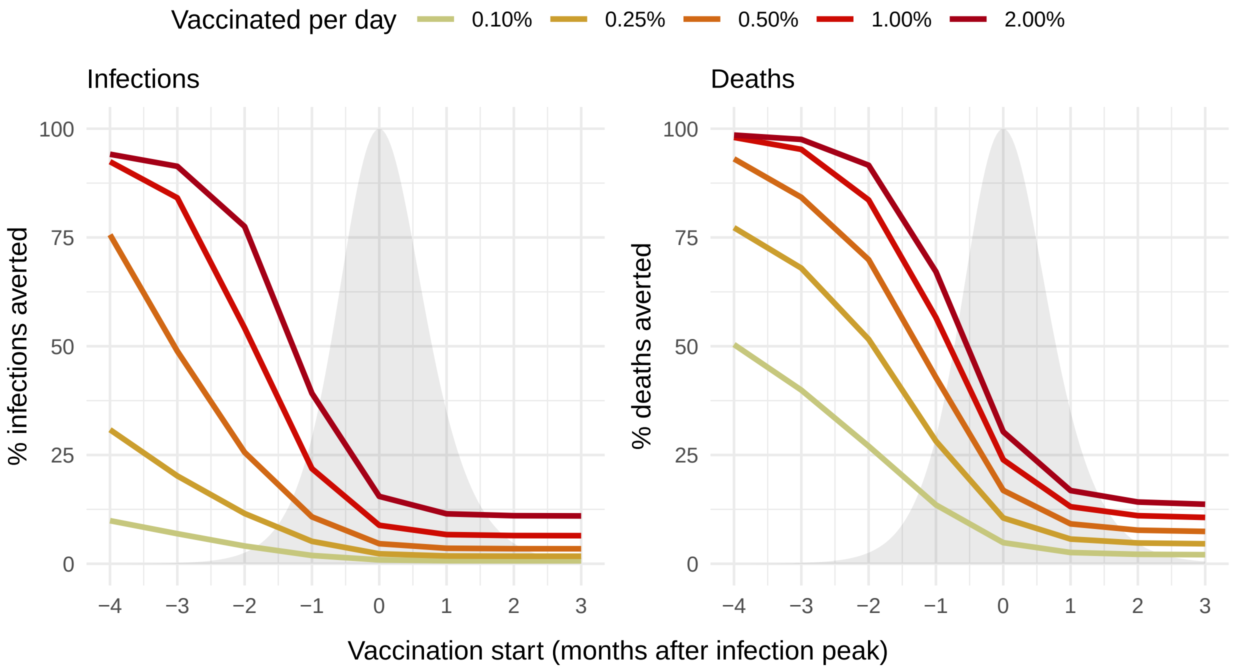

Figure S6: Burden Averted Varying Start of Vaccine Campaign. Simulation results for burden averted relative to no vaccine with vaccination campaign having start dates before or after infection peak (horizontal axis). Different curves correspond to different vaccination speeds. Maintains baseline assumptions including 95% efficacious vaccine. Results consider a fast growth epidemic scenario.

Table S1: Summary of Clinical Trials for COVID-19 Vaccines.*Trial reports immunogenicity at least three weeks after dose administration, before a second dose (if planned) has been administered, and has a comparable outcome (in terms of age group, dose, and day measured) for second doses. **Treatment arms also included groups in which each vaccine dose arm was administered with and without 50μg of Matrix-M adjuvant.

Vaccine	Phase	Date posted	1st dose results*	Doses	Dose interval (days)	Treatment arms	Age group	N	Clinical trials.gov number
ChAdOx1 nCoV-19	1/2	3/27/2020	Yes	2	28, 56	• 1× 5e10 v.p.<br>• 2× 5e10<br>• 5e10, 2.2e10<br>• 5e10, 3.5-6.5e10	18-55	1090	NCT04324606
	1/2	6/23/2020	No	2	28	2× 5-7.5e10 v.p.	18-65	2130	NCT04444674
	2/3	5/26/2020	Yes	2	28-42	• 1× 5e10 v.p.<br>• 2× 3.5-6.5e10<br>• 5e10, 2.2e10<br>• 5e10, 3.5-6.5e10	18-70+	12390	NCT04400838
	3	9/2/2020	Yes	2	28-84	• 1× 5e10 v.p.<br>• 5-10, 3.5-6.5e10	18+	10300	NCT04536051
JNJ-78436735	1	6/18/2020	Yes	1	n/a	• 1× 5e10 v.p.<br>• 1× 1e11	18-55	25	NCT04436276
	1/2a	6/18/2020	Yes	2	56	• 1× 5e10 v.p.<br>• 2× 5e10<br>• 1× 1e11<br>• 2× 1e11	18-55, 65+	1085	NCT04436276
	3	8/10/2020	Yes	1	n/a	1× 5e10 v.p.	18+	44325	NCT04505722
mRNA-1273	1	2/25/2020	No	2	28	• 2× 25μg<br>• 2× 50<br>• 2× 100<br>• 2× 250	18+	120	NCT04283461
	2a	5/28/2020	No	2	28	• 2× 50μg<br>• 2× 100	18+	660	NCT04405076
NVX-CoV2373**	1/2	4/30/2020	Yes	2	21	• 2× 5μg<br>• 2× 25<br>• 5, 25	18-59/ 18-84	131/ 1500	NCT04368988
BNT162b2	1	4/20/2020	No	2	21	• 2× 10μg<br>• 2× 20<br>• 2× 30<br>• 2× 100	18-55, 65-85	195	NCT04368728
	2/3	4/20/2020	No	2	21	30μg	12-15, 16-55, 55+	43548	NCT04368728

Table S2: Immunogenicity Data from Phase 1/2 Trials. Neutralizing antibody (nAb) responses listed are the peak levels recorded in published trial data. Sources: ChAdOx1 nCoV-19 (Oxford/AstraZeneca) (*14*), BBV152 (COVAXin) (*34*), JNJ-78436735 (Johnson & Johnson) (*67*), mRNA-1273 (Moderna) - 25 µg (*68*), mRNA-1273 (Moderna) - 50µg (*33*), NVX-CoV2373 (Novavax) (*69*), BNT162b2 (Pfizer) (*70*). *"Standard dose" is what is being used in vaccine roll-outs thus far (v.p.=viral particles). **Both doses were administered together with 50µg of adjuvant. ***Sample size was too small to determine significance.

Vaccine	nAb assay	Day of 2nd dose	Age group	N	Measured on day	Standard dose*	Dose tested	Dose fraction	NAb response	Standard dose NAb response	NAb response as fraction of standard dose	Difference is significant (at 95% level)?
ChAdOx1 nCoV-19	MN_{80}	14	18-55	41	42	5e10 v.p.	2.2e10 v.p.	0.44	161	193	0.83	no
			56-69	28	42			0.44	143	144	0.99	no
			70+	34	42			0.44	150	161	0.93	no
BBV152	$PRNT_{50}$	28	12-65	190	56	6µg	3µg	0.5	100	197	0.51	yes
JNJ-78436735	PRNT IC_{50}	n/a	18-55	24	56	5e10 v.p.	1e11 v.p.	2	310	288	1.08	no
			65+	50	28			2	212	277	0.77	no
mRNA-1273	$PRNT_{80}$	28	18-55	15	42	100µg	25µg	0.25	340	654	0.52	no
	MN_{50}		18-55	78	42		50µg	0.5	1733	1909	0.91	no
			55+	63	42		50µg	0.5	1827	1686	1.08	no
NVX-CoV2373**	MN $IC_{>99}$	21	18-59	50	35	5µg	25µg	5	3305	3906	0.85	no
BNT162b2***	$PRNT_{50}$	21	18-55	12	28	30µg	10µg	0.33	157	361	0.43	n/a
			65-85	12	35		10µg	0.33	111	206	0.54	n/a
			18-55	12	28		20µg	0.67	363	361	1.01	n/a
			65-85	12	28		20µg	0.67	84	206	0.41	n/a

Table S3: Increase in Vaccine Supply from Fractional Doses. Panel 1 shows the total supply projected for 2021. Panel 2 shows the number of doses already delivered by July 2021. Based on the previous values, panel 3 shows the projected baseline supply per month for the remaining 4 months of the year. Finally, panel 4 shows the size of the alternative dose relative to the standard used to estimate the number of extra doses shown in panel 5, where we assume fractional doses would be adopted starting in October.

**BNT162b2 (Pfizer)**	**mRNA-1273 (Moderna)**	**ChAdOx1 nCoV-19 (Oxford/AstraZeneca)**	**Total**
**1. Projected Supply in 2021 (billions of doses)**			
3.00	0.80	2.10	5.90
**2. Doses Delivered by September 2021**			
1.30	0.20	1.10	2.60
**3. Projected Baseline Monthly Supply (billion doses/month) =** $\frac{[1]-[2]}{4}$			
0.43	0.15	0.25	0.83
**4. Dose Regimen (relative to standard)**			**5. Extra Doses (billions/month)**
1/3	1/4	1/2	1.55
2/3	1/2	1/2	0.61
2/3	1/2	3/4	0.45

Table S4: Comparison of Efficacy and Effectiveness Data for COVID-19 Vaccines. Values represent point estimates of efficacy and effectiveness. Efficacy against symptomatic infection is derived from phase 2/3 trials data. Estimates of effectiveness against symptomatic infection, hospitalization and death come from various observational studies. Viral variants referred to as "non-VOC" includes all variants that are not classified as Variant of Concern, therefore excluding B.1.617.2, B.1.1.7, and B.1.351.
*Study combines data from individuals vaccinated with both BNT162b2 and mRNA-1273.
**Estimates include occurrences of either hospitalization or death.
***Estimates include occurrences of any severe, critical or fatal disease.

**Vaccine**	**Efficacy**		**Effectiveness**					
	Symptomatic Infection	Source	Location	Variant	Symptomatic Infection	Hospitalization	Death	Source
ChAdOx1 nCoV-19	64%	(*71*)	UK	B.1.1.7	75%			(*11*)
				B.1.617.2	67%			
				B.1.1.7		86%		(*12*)
				B.1.617.2		92%		
			Scotland	B.1.1.7	81%			(*38*)
				B.1.617.2	61%			
CoronaVac	51%	(*72*)	Chile	Various	66%	88%	86%	(*13*)
	84%	(*73*)						
BNT162b2	95%	(*36*)	UK	B.1.1.7	94%			(*11*)
				B.1.617.2	88%			
				B.1.1.7		95%		(*12*)
				B.1.617.2		96%		
			Scotland	B.1.1.7	92%			(*38*)
				B.1.617.2	83%			
			Israel	Various	97%	97%	97%	(*74*)
			Qatar	B.1.1.7	90%	100%	100%	(*75*)
				B.1.351	75%	100%	100%	
mRNA-1273	94%	(*76*)	USA	Various	94%			(*77*)*
			Canada	B.1.1.7	91%	94%**		(*78*)
				Non-VOC	91%	96%**		(*78*)
			Qatar	Various	99%	96%***		(*79*)
				B.1.617.2	86%	100%	100%	(*80*)

References

1. T. V. Padma, “Covid vaccines to reach poorest countries in 2023 — despite recent pledges”, (2021; https://www.nature.com/articles/d41586-021-01762-w).
2. J. C. Castillo *et al.*, Market design to accelerate COVID-19 vaccine supply. *Science* **371**, 1107–1109, ISSN: 0036-8075, eprint: https://science.sciencemag.org/content/371/6534/1107.full.pdf, (https://science.sciencemag.org/content/371/6534/1107) (2021).
3. World Health Organization, Weekly epidemiological record. *Weekly Epidemiological Record* **92**, 437–452 (2017).
4. E. C. Wall *et al.*, Neutralising antibody activity against SARS-CoV-2 VOCs B.1.617.2 and B.1.351 by BNT162b2 vaccination. *The Lancet* **397**, 2331–2333 (2021).
5. Public Health England, “Confirmed cases of COVID-19 variants identified in UK”, (2021; https://www.gov.uk/government/news/confirmed-cases-of-covid-19-variants-identified-in-uk).
6. A. Ahuja *et al.*, Preparing for a pandemic: accelerating vaccine availability. *AEA Papers and Proceedings* **111**, 331–35, (https://www.aeaweb.org/articles?id=10.1257/pandp.20211103) (May 2021).
7. J. Miller, “Moderna plans mix of COVID-19 vaccine doses with new Lonza deal”, (2021; https://www.reuters.com/business/healthcare-pharmaceuticals/lonza-add-dutch-production-line-boost-moderna-covid-19-vaccine-2021-06-02/2021).
8. K. Wu *et al.*, Preliminary analysis of safety and immunogenicity of a SARS-CoV-2 variant vaccine booster. *medRxiv*, eprint: https://www.medrxiv.org/content/early/2021/05/06/2021.05.05.21256716.full.pdf, (https://www.medrxiv.org/content/early/2021/05/06/2021.05.05.21256716) (2021).
9. M. Erman, A. Banerjee, “Pfizer to test COVID-19 vaccine in larger group of children below 12”, (2021; https://www.reuters.com/business/healthcare-pharmaceuticals/pfizer-start-large-study-test-covid-19-vaccine-children-below-12-2021-06-08/).
10. D. S. Khoury *et al.*, Neutralizing antibody levels are highly predictive of immune protection from symptomatic SARS-CoV-2 infection. *Nature Medicine* (2021).
11. J. Lopez Bernal *et al.*, Effectiveness of Covid-19 vaccines against the B.1.617.2 (Delta) variant. *New England Journal of Medicine* **385**, 585–594, eprint: https://doi.org/10.1056/NEJMoa2108891, (https://doi.org/10.1056/NEJMoa2108891) (2021).
12. J. Stowe *et al.*, Effectiveness of COVID-19 vaccines against hospital admission with the Delta (B.1.617.2) variant. (https://khub.net/documents/135939561/479607266/Effectiveness+of+COVID-19+vaccines+against+hospital+admission+with+the+Delta+%5C%28B.1.617.2%5C%29+variant.pdf/1c213463-3997-ed16-2a6f-14e5deb0b997?t=1623689315431) (2021).
13. A. Jara *et al.*, Effectiveness of an inactivated sars-cov-2 vaccine in chile. *New england journal of medicine*, eprint: https://doi.org/10.1056/NEJMoa2107715, (https://doi.org/10.1056/NEJMoa2107715) (2021).

14. M. N. Ramasamy *et al.*, Safety and immunogenicity of ChAdOx1 nCoV-19 vaccine administered in a prime-boost regimen in young and old adults (COV002): a single-blind, randomised, controlled phase 2/3 trial. *The Lancet* **396**, 1979–1993 (2020).

15. J. S. S. Arce *et al.*, COVID-19 vaccine acceptance and hesitancy in low- and middle-income countries. *Nature Medicine* **27**, 1385–1394 (2021).

16. Word Health Organization, "WHO Director-General's opening remarks at the media briefing on COVID-19 - 4 August 2021", (2021; https://www.who.int/director-general/speeches/detail/who-director-general-s-opening-remarks-at-the-media-briefing-on-covid-4-august-2021).

17. The White House, "Press briefing by white house COVID-19 response team and public health officials", (2021; https://www.whitehouse.gov/briefing-room/press-briefings/2021/08/18/press-briefing-by-white-house-covid-19-response-team-and-public-health-officials-50/).

18. M. Savage, "UK scientists look at reducing boosters to save vaccine for rest of the world", (2021; https://www.theguardian.com/world/2021/aug/22/uk-scientists-look-at-halving-boosters-to-save-vaccine-for-rest-of-the-world).

19. Word Health Organization, "Interim statement on dose-sparing strategies for COVID-19 vaccines (fractionated vaccine doses)", (2021; https://www.who.int/news/item/10-08-2021-interim-statement-on-dose-sparing-strategies-for-covid-19-vaccines-(fractionated-vaccine-doses)).

20. G. V. Roozen *et al.*, Tolerability, safety and immunogenicity of intradermal delivery of a fractional dose mRNA-1273 SARS-CoV-2 vaccine in healthy adults as a dose sparing strategy. *medRxiv*, eprint: https://www.medrxiv.org/content/early/2021/07/28/2021.07.27.21261116.full.pdf, (https://www.medrxiv.org/content/early/2021/07/28/2021.07.27.21261116) (2021).

21. J. Mateus *et al.*, Low dose mRNA-1273 COVID-19 vaccine generates durable T cell memory and antibodies enhanced by pre-existing crossreactive T cell memory. *medRxiv* (2021).

22. F. Krammer, A correlate of protection for sars-cov-2 vaccines is urgently needed. *Nature Medicine* (2021).

23. Universidade Federal do Espírito Santo, "Pesquisa em viana vai avaliar eficácia de imunização com dose reduzida da vacina astrazeneca", (2021; https://www.ufes.br/conteudo/pesquisa-em-viana-vai-avaliar-eficacia-de-imunizacao-com-dose-reduzida-da-vacina).

24. ClinicalTrials.gov, "COVID-19: safety and immunogenicity of a reduced dose of the BioNTech/Pfizer BNT162b2 vaccine (REDU-VAC)", (2021; https://clinicaltrials.gov/ct2/show/NCT04852861).

25. UK Department of Health and Social Care, "World-first COVID-19 vaccine booster study launches in UK", (2021; https://www.gov.uk/government/news/world-first-covid-19-vaccine-booster-study-launches-in-uk).

26. S. Kominers, A. Tabarrok, "The case for a COVID vaccine lottery", (2020; https://www.bloomberg.com/opinion/articles/2020-11-09/covid-19-vaccine-lottery-is-a-winning-strategy).

27. J. Haushofer, C. J. E. Metcalf, Which interventions work best in a pandemic? *Science* **368**, 1063–1065, ISSN: 0036-8075, eprint: https://science.sciencemag.org/content/368/6495/1063.full.pdf, (https://science.sciencemag.org/content/368/6495/1063) (2020).

28. UK Department of Health, "Statement from the UK Chief Medical Officers on the prioritisation of first doses of COVID-19 vaccines", (2020; https://www.health-ni.gov.uk/news/statement-uk-chief-medical-officers-prioritisation-first-doses-COVID-19-vaccines).

29. J. M. Dan *et al.*, Immunological memory to SARS-CoV-2 assessed for up to 8 months after infection. *Science* **371** (2021).

30. L. B. Rodda *et al.*, Functional SARS-CoV-2-Specific immune memory persists after mild COVID-19. *Cell* **184**, 169–183.e17, ISSN: 0092-8674, (https://www.sciencedirect.com/science/article/pii/S0092867420315658) (2021).

31. J. A. Juno *et al.*, Humoral and circulating follicular helper T cell responses in recovered patients with COVID-19. *Nature medicine* **26**, 1428–1434 (2020).

32. C. Rydyznski Moderbacher *et al.*, Antigen-specific adaptive immunity to SARS-CoV-2 in acute COVID-19 and associations with age and disease severity. *Cell* **183**, 996–1012.e19, ISSN: 0092-8674, (https://www.sciencedirect.com/science/article/pii/S0092867420312356) (2020).

33. L. Chu *et al.*, A preliminary report of a randomized controlled phase 2 trial of the safety and immunogenicity of mRNA-1273 SARS-CoV-2 vaccine. *Vaccine* **39**, 2791–2799, ISSN: 0264-410X, (https://www.sciencedirect.com/science/article/pii/S0264410X21001535) (2021).

34. R. Ella *et al.*, Safety and immunogenicity of an inactivated SARS-CoV-2 vaccine, BBV152: interim results from a double-blind, randomised, multicentre, phase 2 trial, and 3-month follow-up of a double-blind, randomised phase 1 trial. *The Lancet Infectious Diseases* **21**, 950–961, (https://doi.org/10.1016/S1473-3099(21)00070-0) (2021).

35. L. R. Baden *et al.*, Efficacy and safety of the mrna-1273 sars-cov-2 vaccine. *New England Journal of Medicine* **384**, PMID: 33378609, 403–416, eprint: https://doi.org/10.1056/NEJMoa2035389, (https://doi.org/10.1056/NEJMoa2035389) (2021).

36. F. P. Polack *et al.*, Safety and efficacy of the bnt162b2 mrna covid-19 vaccine. *New England Journal of Medicine* **383**, PMID: 33301246, 2603–2615, eprint: https://doi.org/10.1056/NEJMoa2034577, (https://doi.org/10.1056/NEJMoa2034577) (2020).

37. E. C. Wall *et al.*, AZD1222-induced neutralising antibody activity against SARS-CoV-2 Delta VOC. *The Lancet* (2021).

38. A. Sheikh, J. McMenamin, B. Taylor, C. Robertson, Public Health Scotland and the EAVE II Collaborators, SARS-CoV-2 Delta VOC in Scotland: demographics, risk of hospital admission, and vaccine effectiveness. *The Lancet*, 2461–2462 (2021).

39. M. Wadman, "Could too much time between doses drive the coronavirus to outwit vaccines?", (2021; https://www.sciencemag.org/news/2021/01/could-too-much-time-between-doses-drive-coronavirus-outwit-vaccines).

40. C. M. Saad-Roy *et al.*, Epidemiological and evolutionary considerations of SARS-CoV-2 vaccine dosing regimes. *Science* **372**, Publisher: American Association for the Advancement of Science, 363–370, (2021; https://www.science.org/doi/10.1126/science.abg8663) (Apr. 2021).

41. W. P. Hanage, C. A. Russell, Partial immunity and SARS-CoV-2 mutations. *Science* **372**, Publisher: American Association for the Advancement of Science, 354–354, (2021; https://www.science.org/doi/10.1126/science.abi4727) (Apr. 2021).

42. S. Cobey, D. B. Larremore, Y. H. Grad, M. Lipsitch, Concerns about SARS-CoV-2 evolution should not hold back efforts to expand vaccination. *Nature Reviews Immunology*, Publisher: Nature Publishing Group, 330–335, ISSN: 1474-1741, (2021; https://www.nature.com/articles/s41577-021-00544-9) (Apr. 2021).

43. M. Bushman, R. Kahn, B. P. Taylor, M. Lipsitch, W. P. Hanage, "Population impact of SARS-CoV-2 variants with enhanced transmissibility and/or partial immune escape", en, tech. rep., Company: Cold Spring Harbor Laboratory Press Distributor: Cold Spring Harbor Laboratory Press Label: Cold Spring Harbor Laboratory Press Type: article, p. 2021.08.26.21262579, (2021; https://www.medrxiv.org/content/10.1101/2021.08.26.21262579v1).

44. New and Emerging Respiratory Virus Threats Advisory Group, "SARS-CoV-2 immunity-escape variants" (New and Emerging Respiratory Virus Threats Advisory Group, Jan. 2021), (https://assets.publishing.service.gov.uk/government/uploads/system/uploads/attachment_data/file/954990/s1015-sars-cov-2-immunity-escape-variants.pdf).

45. T.-Y. Yeh, G. P. Contreras, "Full vaccination against COVID-19 suppresses SARS-CoV-2 delta variant and spike gene mutation frequencies and generates purifying selection pressure", en, tech. rep., Company: Cold Spring Harbor Laboratory Press Distributor: Cold Spring Harbor Laboratory Press ISSN: 2126-1768 Label: Cold Spring Harbor Laboratory Press Type: article, p. 2021.08.08.21261768, (2021; https://www.medrxiv.org/content/10.1101/2021.08.08.21261768v3).

46. W. O. Kermack, A. G. McKendrick, Contributions to the mathematical theory of epidemics–I. 1927. *Bulletin of Mathematical Biology* **53**, 33–55, ISSN: 0092-8240 (1991).

47. R. M. Anderson, R. M. May, *Infectious diseases of humans: dynamics and control* (Oxford University Press, Oxford, New York, Aug. 1992), ISBN: 9780198540403.

48. E. C. Sabino *et al.*, Resurgence of COVID-19 in Manaus, Brazil, despite high seroprevalence. *The Lancet* **397**, 452–455, ISSN: 01406736 (Feb. 2021).

49. N. G. Davies *et al.*, Age-dependent effects in the transmission and control of COVID-19 epidemics. en, *Nature Medicine*, 1–7, ISSN: 1546-170X, (2020; https://www.nature.com/articles/s41591-020-0962-9) (June 2020).

50. E. Goldstein, M. Lipsitch, M. Cevik, On the effect of age on the transmission of SARS-CoV-2 in households, schools, and the community. *The Journal of Infectious Diseases*, jiaa691, ISSN: 0022-1899, 1537-6613, (2021; https://academic.oup.com/jid/advance-article/doi/10.1093/infdis/jiaa691/5943164) (Oct. 2020).

51. J. Mossong *et al.*, Social contacts and mixing patterns relevant to the spread of infectious diseases. en, *PLOS Medicine* **5**, e74, ISSN: 1549-1676, (2019; https://journals.plos.org/plosmedicine/article?id=10.1371/journal.pmed.0050074) (Mar. 2008).

52. D. Manheim, W. Więcek, V. Schmit, J. Morrison, 1Day Sooner Research Team, Exploring risks of human challenge trials for COVID-19. en, *medRxiv*, Publisher: Cold Spring Harbor Laboratory Press, 2020.11.19.20234658, (2021; https://www.medrxiv.org/content/10.1101/2020.11.19.20234658v1) (Nov. 2020).

53. E. Mathieu *et al.*, A global database of COVID-19 vaccinations. *Nature Human Behaviour* (2021).

54. Airfinity Ltd, "COVID-19 vaccine stock forecast for 2021 and 2022", (2021; https://assets.ctfassets.net/poihmvxzgivq/O73jKtn79aS1gPKte1wJZ/74cf192bd3efaee67b93451ba3e9b340/Airfiinty-s_Vaccine_Stock_Forecast_September_2021.pdf).

55. M. Monod *et al.*, Age groups that sustain resurging COVID-19 epidemics in the united states. *Science*, (https://science.sciencemag.org/content/371/6536/eabe8372) (Mar. 2021).

56. Pfizer Inc., "Pfizer and BioNTech to provide 500 million doses of COVID-19 vaccine to U.S. government for donation to poorest nations", (2021; https://investors.pfizer.com/investor-news/press-release-details/2021/Pfizer-and-BioNTech-to-Provide-500-Million-Doses-of-COVID-19-Vaccine-to-U.S.-Government-for-Donation-to-Poorest-Nations/default.aspx).

57. Moderna, Inc., "Moderna announces additional investments to increase global supply for COVID-19 vaccine to up to 3 billion doses in 2022", (2021; https://investors.modernatx.com/news-releases/news-release-details/moderna-announces-additional-investments-increase-global-supply).

58. Duke Global Health Innovation Center, "Launch & scale speedometer", (2021; https://launchandscalefaster.org/covid-19/vaccinemanufacturing).

59. C. Zimmer, J. Corum, S.-L. Wee, "Coronavirus vaccine tracker", (2021; https://www.nytimes.com/interactive/2020/science/coronavirus-vaccine-tracker.html).

60. Pfizer, Inc., "Pfizer and biontech announce collaboration with brazil's eurofarma to manufacture covid-19 vaccine doses for latin america", (2021; https://investors.pfizer.com/investor-news/press-release-details/2021/Pfizer-and-BioNTech-Announce-Collaboration-With-Brazils-Eurofarma-to-Manufacture-COVID-19-Vaccine-Doses-for-Latin-America/default.aspx).

61. Moderna, Inc., "Joint statement from moderna and takeda", (2021; https://investors.modernatx.com/news-releases/news-release-details/joint-statement-moderna-and-takeda/).

62. AstraZeneca PLC, "Astrazeneca and european commission reach settlement agreement over vaccine supply, ending litigation", (2021; https://www.astrazeneca.com/content/astraz/media-centre/press-releases/2021/astrazeneca-and-european-commission-reach-settlement-agreement-over-vaccine-supply-ending-litigation.html).

63. UNICEF, "COVID-19 vaccine market dashboard", (2021; https://www.unicef.org/supply/covid-19-vaccine-market-dashboard).

64. Multilateral Leaders Task Force on COVID-19, "Task force on covid-19 vaccines, therapeutics, and diagnostics", (2021; https://data.covid19taskforce.com/data).

65. International Monetary Fund, "Secured vaccines and/or expected vaccine supply", (2021; https://www.imf.org/en/Topics/imf-and-covid19/IMF-WHO-COVID-19-Vaccine-Supply-Tracker).

66. Airfinity Ltd, "COVID-19 vaccine expiry forecast for 2021 and 2022", (2021; https://assets.ctfassets.net/poihmvxzgivq/6pooxWkcaF1bHmlP7M5dtP/d4bdc2c7707f4a4ad56b7e967063fa84/Vaccine_expiry_forecast_airfinity_21.09.20.pdf).

67. J. Sadoff *et al.*, Interim results of a phase 1–2a trial of Ad26.COV2.S COVID-19 vaccine. *New England Journal of Medicine* **384**, PMID: 33440088, 1824–1835, eprint: https://doi.org/10.1056/NEJMoa2034201, (https://doi.org/10.1056/NEJMoa2034201) (2021).

68. L. A. Jackson *et al.*, An mRNA vaccine against SARS-CoV-2 — preliminary report. *New England Journal of Medicine* **383**, PMID: 32663912, 1920–1931, eprint: https://doi.org/10.1056/NEJMoa2022483, (https://doi.org/10.1056/NEJMoa2022483) (2020).

69. C. Keech *et al.*, Phase 1–2 trial of a SARS-CoV-2 recombinant spike protein nanoparticle vaccine. *New England Journal of Medicine* **383**, PMID: 32877576, 2320–2332, eprint: https://doi.org/10.1056/NEJMoa2026920, (https://doi.org/10.1056/NEJMoa2026920) (2020).

70. E. E. Walsh *et al.*, Safety and immunogenicity of two RNA-based COVID-19 vaccine candidates. *New England Journal of Medicine* **383**, 2439–2450, eprint: https://doi.org/10.1056/NEJMoa2027906, (https://doi.org/10.1056/NEJMoa2027906) (2020).

71. M. Voysey *et al.*, Safety and efficacy of the ChAdOx1 nCoV-19 vaccine (AZD1222) against SARS-CoV-2: an interim analysis of four randomised controlled trials in Brazil, South Africa, and the UK. en, *The Lancet* **397**, 99–111, ISSN: 0140-6736, (2021; http://www.sciencedirect.com/science/article/pii/S0140673620326611) (Jan. 2021).

72. R. Palacios *et al.*, Efficacy and safety of a COVID-19 inactivated vaccine in healthcare professionals in brazil: the PROFISCOV study. (2021).

73. M. D. Tanriover *et al.*, Efficacy and safety of an inactivated whole-virion SARS-CoV-2 vaccine (CoronaVac): interim results of a double-blind, randomised, placebo-controlled, phase 3 trial in Turkey. *The Lancet* **398**, 213–222, ISSN: 0140-6736 (2021).

74. E. J. Haas *et al.*, Impact and effectiveness of mRNA BNT162b2 vaccine against SARS-CoV-2 infections and COVID-19 cases, hospitalisations, and deaths following a nationwide vaccination campaign in Israel: an observational study using national surveillance data. *The Lancet* **397**, 1819–1829 (2021).

75. L. J. Abu-Raddad, H. Chemaitelly, A. A. Butt, Effectiveness of the BNT162b2 Covid-19 vaccine against the B.1.1.7 and B.1.351 variants. *New England Journal of Medicine* **385**, PMID: 33951357, 187–189, eprint: https://doi.org/10.1056/NEJMc2104974, (https://doi.org/10.1056/NEJMc2104974) (2021).

76. L. R. Baden *et al.*, Efficacy and Safety of the mRNA-1273 SARS-CoV-2 Vaccine. en, *New England Journal of Medicine*, Publisher: Massachusetts Medical Society, (2021; https://www.nejm.org/doi/10.1056/NEJMoa2035389) (Dec. 2020).

77. T. Pilishvili *et al.*, Interim estimates of vaccine effectiveness of Pfizer-BioNTech and Moderna COVID-19 vaccines among health care personnel—33 US sites, January–March 2021. *Morbidity and Mortality Weekly Report* **70**, 753 (2021).

78. S. Nasreen *et al.*, Effectiveness of COVID-19 vaccines against variants of concern in Ontario, Canada. *medRxiv*, eprint: https://www.medrxiv.org/content/early/2021/07/16/2021.06.28.21259420.full.pdf, (https://www.medrxiv.org/content/early/2021/07/16/2021.06.28.21259420) (2021).
79. H. Chemaitelly *et al.*, mRNA-1273 COVID-19 vaccine effectiveness against the B. 1.1. 7 and B. 1.351 variants and severe COVID-19 disease in Qatar. *Nature medicine*, 1–8 (2021).
80. P. Tang *et al.*, BNT162b2 and mRNA-1273 COVID-19 vaccine effectiveness against the Delta (B.1.617.2) variant in Qatar. *medRxiv*, eprint: https://www.medrxiv.org/content/early/2021/08/11/2021.08.11.21261885.full.pdf, (https://www.medrxiv.org/content/early/2021/08/11/2021.08.11.21261885) (2021).
